# The Spontaneous Regression of Primary Gastrointestinal Malignancies: An Observational Review

**DOI:** 10.7759/cureus.32970

**Published:** 2022-12-26

**Authors:** Carlos D Minacapelli, Philip Leuszkiewicz, Ankoor Patel, Carolyn Catalano, George Abdelsayed, Alexander Lalos, Vinod Rustgi

**Affiliations:** 1 Medicine, Division of Gastroenterology and Hepatology, Rutgers Robert Wood Johnson Medical School, New Brunswick, USA; 2 Medicine, Division of Gastroenterology and Hepatology, Rutgers Robert Wood Johnson Medical School, New Brusnwick, USA; 3 Medicine, Division of Internal Medicine, Rutgers Robert Wood Johnson Medical School, New Brunswick, USA

**Keywords:** hepatobiliary system, neoplasms, oncology, gastroenterology, hepatology, gastrointestinal cancers, hepatocellular carcinoma, spontaneous remission, spontaneous regression

## Abstract

The spontaneous regression or remission (SR) of cancer, often described as the partial or complete disappearance of a malignant tumor in the absence of all medical treatment and therapy, is a well-documented phenomenon. With efforts ongoing to establish cancer treatments that limit undesirable outcomes and adverse effects, these uncommon occurrences of SR carry significant implications for novel therapies and warrant further investigation. While several case studies have reported instances of SR in gastrointestinal (GI) malignancies, a comprehensive review of previous manifestations of SR in the GI tract remains lacking. The inclusion criteria for the rare phenomenon are also in need of an appropriate update that takes recent scientific advancements and emerging new medical technologies into account. Our analysis of 390 cases of SR in the GI tract focuses primarily on neoplasms of the hepatobiliary system and proposes an updated version of the older inclusion criteria for spontaneous regression.

## Introduction and background

In 2021 alone, 372,470 new cases of primary gastrointestinal (GI) cancer were reported worldwide, and approximately 124,348 deaths occurred as a result, comprising 19.6% and 20.4% of total new cancer cases and deaths, respectively [1]. Nonetheless, with the introduction of various novel diagnostic, therapeutic, and antineoplastic modalities, the mortality rates of GI cancers have declined significantly over the past several years [2]. These new modalities have led to a greater understanding of the pathogenesis of cancer and of achieving remission. Spontaneous regression (SR) is defined as the complete or partial disappearance of a primary and/or disseminated lesion of a histologically diagnosed metastatic disease in the absence of any medical treatment or therapy known to have antitumor effects. Spontaneous regression has been found to occur throughout the entire body, including the GI tract [3]. But it is not equivalent to a cure, as cancer may reappear or spread elsewhere in the body. The frequency of spontaneous regression varies based on the type of cancer, as it is most commonly reported in renal cell carcinoma, melanoma, and neuroblastoma [3-5]. Occurring at a rate of about one out of every 60,000 to 100,000 cases of all cancers, these extremely rare occurrences of SR have the potential to serve as an instructive in vivo model of biological tumor regulation and control [6].

A number of putative mechanisms have been proposed for the observed spontaneous disappearance of malignancies, including inflammation, apoptosis, ischemia, and immunological responses [7]. Other mechanisms proposed to cause SR include epigenetic modifications, hormonal responses, oncogenes, tumor suppressors, cytokines and growth factors, and psychological mechanisms. Unfortunately, several of these postulated mechanisms are based on association and speculation alone, with the exact mechanistic modalities surrounding the SR of GI cancer yet to be elucidated. Regardless, an immunological anti-tumoral response of a patient’s body to specific malignancies is among the most prevalently described mechanistic hypotheses for the observed spontaneous disappearance of neoplasms. Since the very inception of the term, spontaneous regression has historically been speculated to be a dynamic interplay of immunological anti-tumor responses. In 1956, Everson and Cole defined the criterion for SR as the partial or complete disappearance of a malignant tumor in the absence of all treatment or in the presence of therapy that is considered inadequate to exert a significant influence on neoplastic disease. At that time, they theorized that the phenomenon must be an opportunistic by-product of an activated immune response. Cases of SR linked to infections have significantly influenced the discovery of several different anticancer therapies that facilitate the targeting of cancer cells by the host’s immune system. For example, immune checkpoint inhibitors have revolutionized modern cancer treatment by targeting inhibitory receptors (e.g., PD-1, CTLA-4, LAG-3), ligands (e.g., PD-L1) expressed on T cells, antigen-presenting cells, and tumor cells, which result in an anti-tumor response by stimulating the host immune system.

Focusing chiefly on malignancies of primary GI origin, this observational review of the literature hopes to bring further attention to the phenomenon of SR while also identifying some potential mechanisms that have been purported to contribute to this largely unreported phenomenon. Secondarily, this comprehensive review aims to introduce a revised and up-to-date version of the older inclusion criteria for SR throughout the body. This updated criterion has been modified in a way that takes into account recent scientific advancements and emerging new medical technologies, with the intent that it will also be easy to follow for physicians and clinical researchers alike. Finally, this study seeks to broaden the scope of how SR is perceived by clinicians and members of the medical community by encouraging a holistic view of the exceptionally rare phenomenon as a dynamic interplay of various modalities.

## Review

Materials and methods: 

Search Strategy 

A literature search across five databases (PubMed, Medline, Google Scholar, Semantic Scholar, and Jstage) was performed employing the following main keywords: gastrointestinal cancer, spontaneous regression, spontaneous remission, spontaneous necrosis, and abscopal effect. A full list of searched keywords is included in Appendix A. The clinical characteristics of each occurrence of SR within the GI pathway and the related long-term outcomes were then extracted. Articles were excluded if any systemic treatment was used or if any treatment directed at the lesion was utilized before the documented regression. All diseases were limited to the GI tract, spanning the oral cavity, esophagus, stomach, liver, bile duct, gallbladder, pancreas, mesenteries, peritoneum, small intestine, colon, and rectum. A manual search of each work’s citations was performed, utilizing additional published works listed in the supplementary materials or reference sections of each of the aforementioned studies. No restriction was applied to the date of publication, the form of publication, or the primary language of the publication.

**Table 1:** Baseline characteristics of spontaneous regression within the study sample

Patient characteristics*	Oral (n=46)	Esophageal (n=11)	Gastric (n=38)	Peritoneal (n=3)	Hepatobiliary (n=212)	Pancreatic (n=10)	Small bowel (n=10)	Colorectal (n=60)	Total (n=390)
Age (Mean (SD))	61.2 (17.4)	59.0 (16.2)	60.1 (19.0)	47.7 (15.9)	64.7 (13.5)	50.4 (18.2)	51 (16.2)	62.1 (15.0)	63.1 (14.7)
Age group (n (%))									
<19	1 (2.2%)	0	1 (2.6%)	0	0	0	0	1 (1.7%)	3 (0.8%)
19-34	3 (6.5%)	1 (9.1%)	2 (5.3%)	0	6 (2.8%)	2 (20.0%)	1 (10.0%)	1 (1.7%)	16 (4.1%)
35-44	1 (2.2%)	1 (9.1%)	7 (18.4%)	2 (66.7%)	6 (2.8%)	1 (10.0%)	2 (20.0%)	7 (11.7%)	27 (6.9%)
45-54	11 (23.9%)	1 (9.1%)	2 (5.3%)	0	21 (9.9%)	1 (10.0%)	1 (10.0%)	7 (11.7%)	44 (11.3%)
55-64	8 (17.4%)	4 (36.4%)	10 (26.3%)	0	49 (23.1%)	2 (20.0%)	5 (50.0%)	17 (28.3%)	95 (24.4%)
65-74	9 (19.6%)	2 (18.2%)	5 (13.2%)	1 (33.3%)	82 (38.7%)	1 (10.0%)	1 (10.0%)	13 (21.7%)	114 (29.2%)
75-84	11 (23.9%)	2 (18.2%)	10 (26.3%)	0	43 (20.3%	1 (10.0%)	0	12 (20.0%)	79 (20.3%)
85+	2 (4.3%)	0	1 (2.6%)	0	4 (1.9%)	0	0	2 (3.3%)	9 (2.3%)
Sex (n (%))									
Male	25 (54.3%)	8 (72.7%)	23 (60.5%)	2 (66.7%)	168 (79.2%)	6 (60.0%)	5 (50.0%)	35 (58.3%)	272 (69.7%)
Female	21 (45.7%)	3 (27.3%)	15 (39.5%)	1 (33.3%)	44 (20.8%)	4 (40.0%)	5 (50.0%)	25 (41.7%)	118 (30.3%)
Site of Regression (n (%))									
Primary tumor/Recurrence	41 (89.1%)	8 (72.7%)	35 (92.1%)	0	194 (91.5%)	10 (100.0%)	7 (70.0%)	48 (80.0%)	288 (73.8%)
Lung metastases	2 (4.3%)	3 (27.3%)	0	1 (33.3%)	28 (13.2%)	0	1 (10.0%)	2 (3.3%)	37 (9.5%)
Liver metastases	0	0	1 (2.6%)	1 (33.3%)	2 (0.9%)	2 (20.0%)	2 (20.0%)	10 (16.7%)	18 (4.6%)
Lymph metastases	5 (10.9%)	2 (18.2%)	1 (2.6%)	0	2 (0.9%)	0	3 (30.0%)	2 (3.3%)	15 (3.8%)
Other metastases	1 (2.2%)	1 (9.1%)	1 (2.6%)	1 (33.3%)	13 (6.1%)	0	1 (10.0%)	6 (10.0%)	24 (6.2%)
Extent of regression (n (%))									
Complete	43 (93.5%)	9 (81.8%)	32 (84.2%)	2 (66.7%)	156 (73.6%)	8 (80.0%)	10 (100.0%)	57 (95.0%)	317 (81.3%)
Partial	3 (6.5%)	2 (18.2%)	6 (15.8%)	1 (33.3%)	56 (26.4%)	2 (20.0%)	0	3 (5.0%)	73 (18.7%)
Histological profile (n (%))									
Carcinoma	11 (23.9%)	8 (72.7%)	8 (21.1%)	0	198 (93.4%)	9 (90.0%)	1 (10.0%)	54 (90.0%)	289 (74.1%)
Primary lymphoma	28 (60.9%)	1 (9.1%)	24 (63.2%)	0	4 (1.9%)	0	6 (60.0%)	4 (6.7%)	67 (17.2%)
NET	1 (2.2%)	0	6 (15.8%)	0	2 (0.9%)	1 (10.0%)	1 (10.0%)	1 (1.7%)	12 (3.1%)
Other	6 (13.0%)	2 (18.2%)	0	3 (100.0%)	8 (3.8%)	0	2 (20.0%)	1 (1.7%)	22 (5.6%)
Period of regression (n (%))									
<1 month	4 (8.7%)	0	3 (7.9%)	0	6 (2.8%)	0	0	0	13 (3.3%)
1-1.5 months	3 (6.5%)	1 (9.1%)	5 (13.2%)	0	11 (5.2%)	1 (10.0%)	0	9 (15.0%)	30 (7.7%)
2-5 months	2 (4.3%)	2 (18.2%)	6 (15.8%)	1 (33.3%)	27 (12.7%)	0	3 (30.0%)	13 (21.7%)	54 (13.8%)
6-11 months	6 (13.0%)	3 (27.3%)	3 (7.9%)	0	16 (7.5%)	1 (10.0%)	3 (30.0%)	1 (1.7%)	33 (8.5%)
12-23 months	6 (13.0%)	2 (18.2%)	5 (13.2%)	0	41 (19.3%)	2 (20.0%)	0	9 (15.0%)	65 (16.7%)
24-35 months	6 (13.0%)	1 (9.1%)	4 (10.5%)	1 (33.3%)	30 (14.2%)	1 (10.0%)	0	3 (5.0%)	46 (11.8%)
36-47 months	5 (10.9%)	0	2 (5.3%)	0	14 (6.6%)	1 (10.0%)	1 (10.0%)	5 (8.3%)	28 (7.2%)
48 months+	8 (17.4%)	1 (9.1%)	8 (21.1%)	1 (33.3%)	33 (15.6%)	4 (40.0%)	2 (20.0%)	17 (28.3%)	74 (19.0%)
Unspecified	6 (13.0%)	1 (9.1%)	2 (5.3%)	0	34 (16.0%)	0	1 (10.0%)	3 (5.0%)	47 (12.1%)
Malignancy recurrence (n (%))									
Reported	5 (10.9%)	0	1 (2.6%)	0	14 (6.6%)	1 (10.0%)	2 (20.0%)	1 (1.7%)	24 (6.2%)
Not reported	41 (89.1%)	11 (100.0%)	37 (97.4%)	3 (100.0%)	198 (93.4%)	9 (90.0%)	8 (80.0%)	59 (98.3%)	366 (93.8%)

Inclusion Criteria

**Figure 1:**
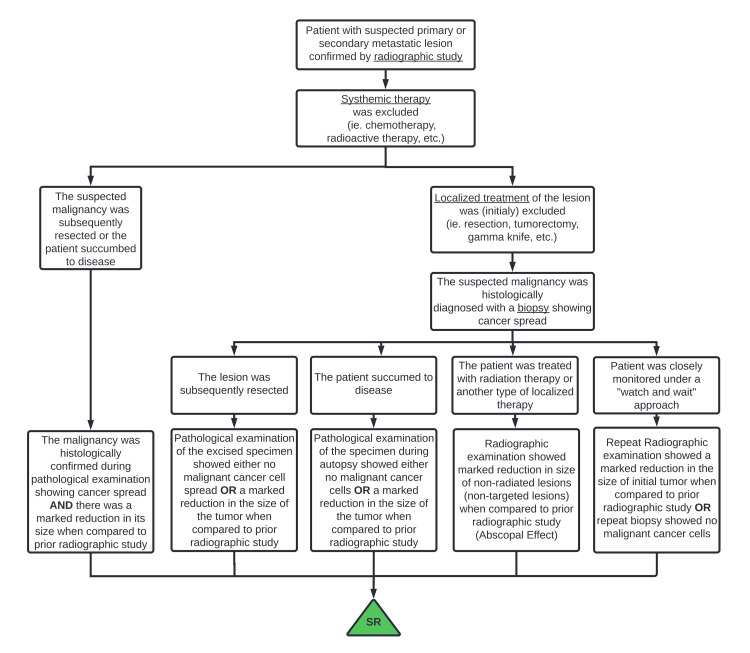
Clinician guidelines or criteria for reporting spontaneous regression From a clinician’s initial encounter with a patient with a suspected cancerous lesion to the demonstration of tumor shrinkage or disappearance, potential clinical manifestations of spontaneous regression are schematically investigated and shown. Original figure by the authors.

Only publications that described the true SR of a histologically confirmed GI cancer were included following the inclusion criteria depicted in Figure 1. These criteria are based on the original criteria proposed by Cole, modified to emphasize histological diagnosis, and adapted to fit multiple clinical scenarios (2). These criteria are summarized as follows: (1) Partial or complete disappearance of the primary tumor or secondary metastasis was radiographically or pathologically demonstrated in the absence of systemic therapy; (2) localized therapy to the lesion prior to the observed shrinkage was excluded; and (3) the malignant neoplasm was histologically proven at some point during this course. Patients with primary neoplasms histologically determined to have originated from outside the GI pathway but demonstrating SR were excluded, even if they demonstrated SR of a secondary metastasis within the GI tract. Patients demonstrating regression of an extra-digestive lesion, histologically determined to have arisen in the GI tract, were included regardless of whether the primary GI lesion had also regressed.

Data Extraction and Analysis

The following information was extracted and recorded from each article: patient age and sex, location and histological typing of the primary tumor, the site of regression, the period of regression or remission, and the etiological mechanism of regression proposed by the author. The demonstrated recurrence of cancer was also noted in some patients. Limited to each author’s interpretation and the duration of follow-up included in each study, the period of remission was defined as one of the following, whichever was found to be the longest: (1) the total period of time during which the tumor demonstrated a shrinkage in size, beginning with the date when the tumor’s size was found to be at its maximum to the date when the tumor’s size was found to be at its minimum, (2) The total period of time between the partial or complete disappearance of cancer and the most recent follow-up date in which the patient continued to show no signs of metastatic spread or recurrence of the malignancy; (3) the total period of time during which the tumor demonstrated a shrinkage in size prior to its resection, from the proposed date when the tumor’s size was found to be at its maximum size to the date of the resection. After the tumor was resected, the specimen was pathologically found to have shrunk or disappeared.

Results

Of the 390 cases of SR of GI malignancies reported meeting our criteria, a majority were noted in men (272 cases, 69.7%) compared to women (118 cases, 30.3%). The mean patient age was 63 years, with a majority of patients between 65 and 74 years of age (114 cases, 29.2%) or 55 and 64 years of age (95 cases, 24.4%). Overall, the literature search demonstrated a global incidence of SR, with cases spanning all six inhabited continents.

**Figure 2:**
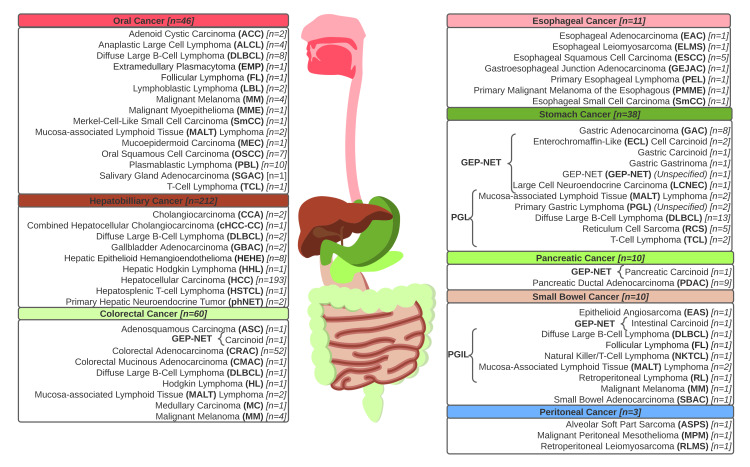
Histological manifestations of gastrointestinal malignancies are recorded to have undergone spontaneous regression throughout the clinical literature. Biopsy-confirmed cancers of the gastrointestinal pathway, including various carcinomas, gastroenteropancreatic neuroendocrine tumors (GEP-NETs), and primary oral and gastrointestinal lymphomas, have been shown to demonstrate spontaneous regression. These cases have been observed throughout the entirety of the alimentary canal, spanning all of the organs of digestion. Cases of each distinct histological denomination were enumerated and systematically organized by the anatomical distribution of the primary lesion. Original figure by the authors.

All reported cases detailing the SR of GI malignancies throughout the clinical literature are comprehensively reviewed in Appendix B, with pertinent findings summarized in Table 1. These reported cases of SR included various cases of carcinoma (289 cases, 74.1%), primary gastrointestinal and oral lymphomas (67 cases, 17.2%), and a few neuroendocrine tumors (12 cases, 3.1%), among other primary gastrointestinal cancers (22 cases, 5.6%). Hepatocellular carcinoma (HCC) represented almost half of all reported cases of SR in GI cancers (193 cases, 49.5%). Several rare forms of cancer, including extramedullary plasmacytoma (EMP), peritoneal alveolar soft-part sarcoma (ASPS), and gastric gastrinoma, were also observed to spontaneously regress. A complete list of reported histological manifestations of GI malignancies recorded to have undergone SR is in Figure 2.

**Table 2:** Proposed mechanisms of spontaneous regression within the gastrointestinal pathway

Proposed mechanism	n (%)
Immunological	202 (51.8%)
Abscopal effect	10 (2.5%)
Endocrine factors	4 (1.0%)
Restored immunogenicity	18 (4.6%)
Eradication of oncogenic virus	7 (1.8%)
Fever/Infection	35 (9.0%)
Inflammatory response	14 (3.6%)
Transfusions	1 (0.3%)
Treatment of primary/Metastases	7 (1.8%)
Other/Not specified	106 (27.2%)
Ischemic	146 (37.4%)
Anti-angiogenic factors	3 (0.8%)
Vascular ischemia/ thrombosis	26 (6.7%)
Tumor ablation/Biopsy/Angiography	44 (11.3%)
Tumor hypoxia/ Hypoperfusion	30 (7.7%)
Tumor microenvironment disruption	3 (0.8%)
Unpredictable/Rapid growth	14 (3.6%)
Other/Not specified	26 (6.7%)
Idiopathic	92 (23.6%)
Apoptotic tumor cell death	6 (1.5%)
Dislodged	10 (2.6%)
Drugs	14 (3.6%)
Genetic	5 (1.3%)
Herbal medicines	20 (5.1%)
Metabolic/Nutritional	7 (1.8%)
Psychoneurological	5 (1.3%)
Withdrawal of carcinogenic agent	16 (4.1%)
Other	9 (2.3%)
Not specified	68 (17.4%)

The vast majority of cases of partial or complete regression occurred within the primary tumor (288 cases, 73.8%); nonetheless, multiple cases demonstrated regression of liver metastases (18 cases, 4.6%), lung metastases (37 cases, 9.5%), lymph metastases (15 cases, 3.8%), or other metastases (24 cases, 6.2%). The period of regression (as defined above) varied greatly in these cases, with some cases reporting just a few days of regression and others expressing several years of remission. Cancer recurrence was reported in 24 cases of SR, comprising 6.2% of total cases of SR in the GI pathway (noted with an asterisk "*" in Appendix B).

The authors proposed various putative mechanisms of SR, which are summarized in Table 2.

The majority of the reviewed authors provided at least one conjectural mechanism (322 cases, 82.6%), with most citing immunological (202 cases, 51.8%), ischemic (146 cases, 37.4%), or idiopathic (92 cases, 23.6%) processes.

Discussion

This systematic review includes the presentation of 390 cases reported in 346 scientific papers, journals, case studies, and published books. These publications were generated over the past 95 years. Interestingly, 325 cases were published in the modern era, defined as cases published in the past 30 years. Following the review of these articles, multiple common factors were revealed, including a tendency for SR to occur in patients over the age of 55 (297 patients, 76.2%), patients of the male sex (272 patients, 69.7%), and patients with primary liver tumors (209 patients, 53.6%) or secondary liver metastases (18 patients, 4.6%). The clinical features and proposed mechanisms surrounding these cases of SR within the GI pathway, along with the location and duration of remission, are documented in Appendix B. Patients within this cohort of reported cases displayed varied periods of stability, ranging from just a few days of observed partial tumor regression to several years of cancer remission, up to 20 years cancer-free.

Mechanisms of Spontaneous Regression

Historically, SR has been speculated to occur in the setting of a prolonged febrile illness due to viral or bacterial infection; nonetheless, only a fraction of cases of SR (35 patients, or 5.1%) have been attributed to a hyperthermic state and infection [8]. In cases of SR occurring during times of acute febrile infections, immune cell infiltrations and signaling cascades are postulated to lead to tumor cell death and cancer tissue necrosis via the release of interleukins, tumor necrosis factors, and interferons (specifically IL-2, IL-6, and IL-8) [9-17]. Viral infections notably induce the production of interferons, which are capable of their own immunomodulatory effects involving macrophages, B-cells, and monocytes, alongside the induction of IL-2 receptors in some cancers [6-8]. Most recently, tumor regression has been reported after COVID-19 vaccination and infection with its wide-ranging pro-inflammatory effects on the host immune system [18-23].

Enhanced antitumoral immunogenicity is proposed to play a profound role in the involution of several GI cancers. In fact, examples displaying the correlation between SR and the elimination of immunodisruptive factors (e.g., medications, viral infection, checkpoint proteins) are perhaps the best evidence supporting the involvement of immunological mechanisms in the achieved SR of GI cancer [24,25]. Tumors have also occasionally been found to regress following systemic or localized treatment for some other disease process. For example, certain localized therapies have been observed to cause tumor regression of both the target lesion and any untreated tumors [26]. Described as the "abscopal effect," this phenomenon is purportedly mediated by a systemic anti-tumor response that follows after receiving radiation therapy for a metastatic lesion or an entirely separate neoplasm. Overall, more than half of the reported cases of SR within the GI tract have been attributed to immunological processes (202 cases, 51.8%), with authors also suggesting the involvement of endocrine factors (four cases, 1.0%) and inflammatory responses (14 cases, 3.6%).

Ischemic models of regression are also proposed to play a key role in the dynamic interplay of antitumoral mechanisms described in the SR of cancer. Tumor cells require an ample supply of blood, so limiting their blood supply and perfusion could intuitively starve the cells to death [27-29]. Consequently, systemic and tumoral hypoperfusion (30 cases, 7.7%), rapid and unpredictable growth (14 cases, 3.6%), anti-angiogenic factors (three cases, 0.8%), and vascular compromise (26 cases, 6.7%) are all theorized to lead to the SR of GI cancer [30-34]. For example, there are multiple cases of SR described as following profound systemic hypoperfusion associated with hemodialysis, surgical invasion, or GI hemorrhage [30-34]. Several reviewed cases of SR (44 patients, 11.3%) have been specifically attributed to diagnostic biopsy procedures alongside tumor ablation and angiographic techniques [3,7,35]. In addition to impairing the adequate delivery of essential nutrients and oxygenation to the remaining (AL3) malignant tissue, these procedures are known to set forth a landslide of tumor-derived antigens into circulation, thus acting as a therapeutic vaccine [4,36].

While endocrinologic mechanisms are largely considered to play a secondary role in the course of tumor regulation, notable hormonal changes are considered possible antecedents to SR [37]. In a case describing a presumed appendiceal neuroendocrine tumor (NET) during pregnancy, Sewpaul et al. observed rapid regression following the patient’s completion of her pregnancy, suggesting that the pregnancy did not worsen the course of the disease but instead may have contributed to tumor regression [38]. Additional influences on the endocrine system by psychological events, such as trauma and stress, suggest that a patient’s psychological status might also influence the course of tumor development. In a case study detailing the SR of one patient’s recurrent oral squamous cell carcinoma (OSCC), Oya et al. describe how the 73-year-old patient was unable to understand the state of his recurrent cancer following cerebral infarction and dementia and postulate how this "unconsciousness" functioned as a preferable psychological condition for tumoral regression [39].

Spontaneous Regression in Cancers of Specific Pathohistology

Hepatocellular carcinoma: While testicular germ cell tumors, neuroblastomas, and renal carcinomas are conventionally the most frequent types of histologically diagnosed tumors presenting this phenomenon, several recent studies report an increasing incidence of SR within the GI pathway, particularly in primary hepatic lesions [6,40]. Correspondingly, we found that HCC was by far the most frequently observed type of cancer within the GI pathway to have undergone SR, with 199 total cases reported in the literature from 1982 to 2021. The reviewed cases proposed several mechanisms surrounding the involution of HCC, primarily citing ischemic and immunological antitumoral models of regression.

To prevent a barrage of immune responses to innocuous materials while still enabling immunity to pathogens, the complex cellular, functional, and molecular modeling of the liver allows for a dynamic, multifaceted approach to immune surveillance that incorporates the tolerogenic organ’s inherently immunosuppressive microenvironment and its distinct hepatic regulatory pathways [41]. It is possible that any manipulation of this multipronged system, such as through the abatement of the tolerogenic characteristics of hepatic APCs or the enhancement of effector lymphocyte function, could potentially have the desired effect of increased anti-tumor activity and tumor regression [42].

Interestingly, several of the changes associated with the SR of the poorly prognosed tumor can also be observed following transarterial chemoembolization (TACE) treatment, thus suggesting that the SR of HCC should, to some degree, involve ischemic processes [43]. Regression of HCC has also been linked to rapid tumor infiltration, in which the notably hypervascular tumor grows more rapidly than its blood supply, leading to local or centralized ischemia, intratumoral bleeding, and hemorrhagic necrosis of the lesion [44]. These distinct immunologic and vascular attributes of the liver combine to form a tumoral environment wherein an intrahepatic malignancy is uniquely positioned to respond to immune and ischemic changes compared to tumors of other organs of the GI tract. Otherwise, abstinence from alcohol, persistent fever, withdrawal from androgens, blood transfusions, and the use of herbal medicines have also been described as leading to the SR of primary hepatic lesions.

Primary oral and gastrointestinal lymphoma: Cases detailing the SR of primary oral and GI lymphomas were observed to span the entirety of the alimentary canal, from several primary extranodal lymphomas of the oral cavity to four cases of rectal lymphoma that regressed spontaneously. Regarding the spontaneous regression of aggressive NHLs of the digestive tract, several cases have been reported demonstrating the spontaneous involution of lymphoma following improved immunological status, particularly in HIV-infected patients receiving antiretroviral therapy [27-32].

While SR is an exceptionally rare occurrence in aggressive lymphomas, such as DLBCL and ALCL, it can occur relatively frequently in low-grade lymphomas such as follicular lymphomas (FLs) and mucosa-associated lymphoid tissue (MALT) lymphomas. Generally, well- or moderately-differentiated forms of cancer are considered low immunogenic tumors due to their limited mutational load and concomitant limited neoantigen expression. In a retrospective analysis of 209 cases of NHL from 1965 until 1978, Gattiker et al. reported the occurrence of SR in 18 out of 140 (12.9%) cases of nodular type malignant lymphoma and 2 out of 69 (2.9%) cases of diffuse type malignant lymphoma [45]. The relationship between gastric mucosa-associated lymphoid tissue (MALT) lymphoma and H. pylori is very well established, and low-grade gastric MALT lymphomas are known to regress following the bacteria’s eradication [46]. This reversible reactivity of low-grade MALT lymphomas to H. pylori infection is a clearly documented phenomenon; hence, cases detailing the regression of low-grade MALT lymphomas involving H. pylori eradication through the use of eradication therapy were excluded from the scope of this careful review.

Pancreatic ductal adenocarcinoma: With only a few cases reported in the literature, pancreatic tumors are seldom known to undergo SR, leaving clinicians skeptical of this lethal tumor’s ability to truly demonstrate involution when left untreated. Despite numerous molecular and immunological approaches, pancreatic cancer is typically poorly responsive to existing chemotherapeutic and immunological antineoplastic agents. This lack of response to immunotherapies is largely due to cancer’s low mutational burden and tendency to favor an immunosuppressive microenvironment characterized by self-isolating dense desmoplastic tissue and an exceptionally low number of infiltrating T cells [47,48]. In a recently published article investigating the possibility of misdiagnosis leading to a presumptive finding of SR in pancreatic cancer, Herreros-Villaneuva et al. emphasized how different types of pancreatic carcinomas must be cautiously distinguished from otherwise benign tumors, insulinomas, and immunoglobulin G4 (IgG4)-associated autoimmune pancreatitis during the process of recording SR [49]. Regardless, four additional cases of pancreatic ductal adenocarcinoma (PDAC) have since been published, citing various multifactorial models of SR, including acute pancreatitis and bacterial or fungal infection in the vicinity of the pancreatic tumor, leading to improved immunogenic tumor presentation [48,50-52].

Colorectal cancer: Like pancreatic cancer, colorectal cancer has long been considered poorly immunogenic, largely based on indirect data from epidemiological studies on the lack of SR in colorectal cancer [53,54]. This lack of immunogenicity in this cancer can be attributed to the failure of tumor-infiltrating lymphocytes to demonstrate substantial lytic activity against cancer cells, as demonstrated in in vitro models [55,56]. While colorectal cancer constitutes more than 15% of all malignancies, it represents less than 2% of all tumors to demonstrate SR [57]. Still, several other rare forms of GI cancer, including Merkel cell-like small cell carcinoma of the parotid gland and multiple gastroenteropancreatic neuroendocrine tumors (GEP-NETs) of the stomach, bile duct, and pancreas, were observed to spontaneously regress.

Strengths and Limitations

While prior retrospective analyses have investigated the incidence of SR for specific cancers and its occurrence within the individual organs of digestion, an observational study of this scope, broadly examining all prior cases of SR throughout the entire GI pathway, has never been published to date. This first-of-its-kind study systematically and thoroughly extracts and organizes information from an array of 390 individual cases of SR within the GI pathway. Although the majority of reports were restricted to the English literature, cases in other languages, including Spanish, Chinese, German, and Japanese, are included in this broad review in order to better demonstrate the global incidence of the otherwise rare phenomenon. Putative mechanisms for SR, including immunological, ischemic, and idiopathic modalities, are also explored and discussed in a detailed manner with the hopes of aiding in an understanding of SR as a dynamic interplay of complex and interconnected antitumoral responses.

In general, SR remains a poorly understood and somewhat vaguely defined phenomenon. Our review has multiple limitations. Recognizing true SR as a host response to specific tumors may continue to be obscured by bias in how the regression is reported. In addition to the possible underreporting of cases of SR by certain physicians, there is also a significant amount of variability in how SR is defined. Distinguishing SR from abscopal effects and tumor regression instigated by eradication therapy remains highly subjective and may result in the misreporting of true spontaneous antitumoral host responses to specific cancers. Overall, the literature is quite heterogeneous, and not every case study reported the duration of follow-up or the duration of remission in a similar manner as would be expected in a retrospective review of this kind.

## Conclusions

SR is an extremely rare occurrence. Nonetheless, certain recurrent patterns in cases of SR, as demonstrated in this review, deserve ample consideration. To better study SR in the future, there must be an emphasis on standardizing how SR is reported. A well-defined registry would also be helpful. Ultimately, this broadly encompassing yet focused assessment is meant to bring attention to the phenomenon of SR and perhaps aid in the investigative efforts in the burgeoning field of immunotherapies.

## Appendices

Appendix A

**Table 3:** Keywords included in search criteria Each word from cohort P was cross-searched with a word or phrase from cohorts 1 or 2a and 2b. Phrases from cohort 1 were searched individually after being paired with a word from cohort P. Phrases searched with words from Cohort 2a were matched with a single word from cohort 2b (if applicable), and this pairing was used across the five databases.

Cohort P	Cohort 1	Cohort 2a	Cohort 2b
Abscopal effect	Alveolar soft-part sarcoma	Anus (anal)	Adenocarcinoma
Spontaneous necrosis	Cholangiocarcinoma	Bile duct (biliary/hepatobiliary)	Anaplastic lymphoma
Spontaneous regression	Epithelioid hemangioendothelioma	Cecum (Ileocecal)	Cancer
Spontaneous remission	Extramedullary plasmacytoma	Colon (colorectal)	Carcinoid
	Gastrinoma	Duodenum (duodenal)	Carcinoma
	Insulinoma	Esophagus (esophageal)	Diffuse large B-cell lymphoma
	Islet cell cancer	Gallbladder	Follicular lymphoma
	Malignant myoepithelioma	Gastroesophageal junction	Hodgkin lymphoma
	Malignant peritoneal mesothelioma	Gastrointestinal	Leiomyosarcoma
	Mucoepidermoid carcinoma	Ileum (ileal)	Lymphoblastic lymphoma
	Pseudomyxoma peritonei	Jejunum (jejunal)	Lymphoma
		Liver (hepatic/hepatocellular)	Malignant melanoma
		Mesentery (mesenteric)	MALT lymphoma
		Omentum (omental)	Natural killer lymphoma
		Oral cavity (oral)	Neuroendocrine tumor
		Pancreas (pancreatic)	Plasmablastic lymphoma
		Peritoneum (peritoneal)	T-cell lymphoma
		Rectum (rectal)	
		Salivary gland (adenoid)	
		Small bowel/intestine (enteric)	
		Tongue	

Appendix B 

**Table 4:** Clinical features of cases demonstrating the spontaneous regression of gastrointestinal cancers

		Ref	Age/ Sex	Location	Pathologic Histology	Site of regression	Period of Remission	Proposed mechanism
Oral cancer	1	Roxburgh (1935)	60s/F	Tongue	OSCC	Primary tumor	7 years	Partial Resection
	2	Grillet et al (1984)	26/M	Parotid gland	ACC	Lung metastases	7 years	Diet
	3	Grillet et al (1984)	53/M	Submandibular gland	ACC	Lung & nasolabial metastases	3 years	Not reported
	4	Grem et al (1986)	54/F	Vallecula	DLBCL	Primary tumor	4 years	Immunological; viral/bacterial infection; Biopsy
	5	Poppema et al (1988)	12/M	Oropharynx	LBL	Primary tumor & cervical lymph node	3 years	Immunological (cytotoxic)
	6	Savarrio et al (1999)	77/M	Soft palate	ALCL	Primary tumor	1 year*	Immunological; Biopsy
	7	King et al (2001)	52/M	Parotid gland	MM	Primary tumor & regional lymph nodes	6 weeks	Immunological
	8	Notani et al (2002)	77/M	Tongue	ALCL (TCL)	Primary & multiple oral recurrences	multiple	Not reported
	9	Koga et al (2003)	78/F	Maxillary mingiva	DLBCL	Primary tumor	3 years	Biopsy
	10	Yamamato et al (2003)	80/F	Maxilla	DLBCL	Primary tumor	1.5 years	Biopsy
	11	Yokoyama et al (2003)	46/F	Hard palate	MALT Lymphoma	Primary tumor	2.5 years	Biopsy
	12	Heibel H et al (2004)	70/M	Mandible	DLBCL	Primary tumor	1.5 years	Biopsy
	13	Lester et al (2004)	50/M	Palate	PBL	Primary tumor	4 months*	Restoration of immune function (HAART)
	14	Sakuma et al (2006)	70/F	Palatine salivary gland	MALT Lymphoma	Primary tumor	3 years	Biopsy; Localized infection
	15	Armstrong et al (2007)	35/M	Maxilla	PBL	Primary tumor (partial)	2 weeks	Restoration of immune function (antiretroviral)
	16	Kurita et al (2007)	67/M	Tongue	OSCC	Cervical lymph node metastases	10 months	Enhanced Apoptosis
	17	Oya andikemura K (2007)	73/M	Tongue	OSCC	Primary tumor	3.5 years	Psychoneurological ("unconsciousness to cancer" S/P cerebral infarct & dementia); Immunological
	18	Rujirojindakul et al (2007)	26/M	Submandibular gland	LBL	Primary tumor	multiple*	Not reported
	19	Daly et al (2008)	56/M	Maxillary gingiva	TCL	Primary tumor	4 years	Biopsy
	20	Mulder et al (2009)	78/M	Parotid gland	Merkel cell-like SmCC	Primary tumor	5 months*	T-cell mediated response triggered by trauma; Apoptosis
	21	Brachet et al (2011)	58/F	Hard palate	DLBCL	Primary tumor	15 days*	Biopsy
	22	Corti et al (2011)	55/F	Oral	PBL	Primary tumor	10 months	Restoration of immune function (antiretroviral cART)
	23	Tamás et al (2011)	66/F	Vallecula/tongue	DLBCL	Primary tumor	7 years	Not reported
	24	Fitzpatrick et al (2012)	88/F	Labial mucosa	ALCL (TCL)	Primary tumor	2 weeks	Biopsy
	25	García-Noblejas et al (2013)	78/F	Buccal mucosa	PBL	Buccal & cervical lymph node lesions	2 years	Restoration of immune function (removal of methotrexate)
	26	Choi et al (2014)	52/F	Buccal mucosa	OSCC	Cervical lymph node metastases	Not reported	Tumor Microenvironment modification
	27	Sousa et al (2014)	62/M	Mouth floor	OSCC	Primary tumor	3 months	Biopsy; Immunological
	28	Cuenca-Jimenez et al (2015)	65/M	Parotid gland	OSCC	Primary tumor	Not reported	Not reported
	29	Igawa et al (2015)	80/M	Maxillary gingiva	PBL	Primary tumor	8 months	Genetic (immunosenescence)
	30	Kaibuchi et al (2015)	87/M	Gingiva	DLBCL	Primary tumor	2.5 years	Biopsy
	31	Gonzalez-Perez et al (2016)	75/M	Mandibular gingiva	EMP	Primary tumor	1.5 years	Immunological (cytokines. Growth factors); Biopsy
	32	Wagner et al (2016)	33/F	Mandibular gingiva	PBL	Primary tumor	1.5 months	Restoration of immune function (HAART)
	33	Daroit et al (2017)	66/F	Maxilla	PBL	Primary tumor	1 year	Restoration of immune function (HAART)
	34	Kitamura et al (2017)	61/M	Maxillary gingiva	PBL	Primary tumor	2 years	Restoration of immune function (antiretroviral)
	35	Rajan & Samant et al (2017)	49/F	Hard palate	MM	Primary tumor	Not reported	Immunological
	36	Yao et al (2017)	80/M	Maxillary gingiva	PBL	Primary tumor	Not reported	Traumatic factors (Biopsy)
	37	Miyagawa et al (2018)	46/M	Upper lip	ALCL	Primary tumor	1 year	Biopsy
	38	Gamarra et al (2018)	50/F	Maxilla	MEC	Primary tumor (partial)	10 years	Not reported
	39	Ono et al (2019)	69/M	Mandibular gingiva	PBL	Primary tumor	2 years	Not reported
	40	Curioni et al (2020)	51/F	Submandibular gland	SGAC	Primary tumor	7 years	Metabolic derangement (hypoglycemia); Immunological
	41	Oliveira et al (2020)	52/F	Hard palate	MM	Primary tumor	6 years	Inflammatory; Immunological
	42	Peeters et al (2020)	59/M	Buccal/Masticator space	FL	Primary tumor	6 months	Biopsy
	43	Aoki et al (2021)	84/M	Maxillary gingiva	DLBCL	Primary tumor	2 years	Biopsy
	44	Lau et al (2021)	66/F	Oropharyngeal tonsil	OSCC	Primary tumor	7 months	Biopsy (Anti-tumoral T-cell response); Hyperthermal state (COVID-19 vaccine)
	45	Ueno et al (2021)	83/F	Mandibular gingiva	MM	Primary tumor (partial)	26 days	Not reported
	46	Sousa et al (2022)	61/F	Parotid gland	MME	Primary tumor	9 months	Inflammatory response (COVID-19 vaccine)
Esophageal cancer	47	Rees et al (1983)	49/M	Lower esophagus	EAC	Lung metastases	1 year	Abscopal effect
	48	Oshwada et al (1990)	78/M	Thoracic esophagus	ESCC	Primary tumor (partial) & pulmonary metastases	7 months	Change in T-cell subsets; surgical trauma
	49	Vergeau et al (1991)	36/M	Mid esophagus	ESCC	Primary tumor (partial)	1 year	Leukocyte infiltration; Inflammation
	50	Hahm et al (1993)	30/M	Thoracic esophagus	PEL	Primary tumor	Not reported	H2-blocker (Cimetidine)
	51	Saruki et al (1994)	82/F	Thoracic esophagus	ESCC	Primary tumor	2 years	Infection (Pneumonia)
	52	Takemura et al (1999)	63/F	Thoracic esophagus	ELMS	Pleural & splenic metastases	10 months	Removal of Primary
	53	Chang (2000)	57/M	Lower esophagus	ESCC	Primary tumor	9 years	Infection (Pneumonia); Inflammation
	54	Kubota et al (2003)	73/M	Thoracic esophagus	SCEC	Primary tumor	1 month	Infection (Hepatitis C)
	55	Hornby et al (2015)	57/M	GEJ	MM	Primary tumor	2 months	Immunological; Occult Primary
	56	Mitchell et al (2021)	58/F	GEJ	GEJAC	Local & supraclavicular lymph metastases	6 months	Not Reported
	57	Kahn et al (2021)	66/M	Lower esophagus	ESCC	Primary tumor	3 months	Immunological (T-cell response)
Stomach cancer	58	Takeuchi et al (1971)	39/M	Corpus-antrum	PGL (RCS)	Primary tumor (partial)	2 months	Not reported
	59	Nakano et al (1972)	55/F	Corpus	PGL (RCS)	Primary tumor (partial)	3 weeks	"malignant cycle" of early gastric cancer
	60	Rosenberg et al (1972)	51/M	Lesser curvature	GAC	Liver metastases	12 years	Fever
	61	Ohashi et al (1973)	42/M	Corpus-antrum	PGL (RCS)	Primary tumor (partial)	1 month	"malignant cycle" of early gastric cancer
	62	Tietjen et al (1974)	60/F	Gastric antrum duodenum	PGL (RCS)	Primary tumor	5 years	"malignant cycle" of early gastric cancer
	63	Yamazaki et al (1974)	39/M	Pyloric antrum	PGL (RCS)	Primary tumor (partial)	20 days	Not reported
	64	Kimura et al (1987)	85/F	Residual stomach	GAC	Primary tumor	Not reported	Not reported
	65	Strauchen et al (1987)	73/M	Pyloric antrum	PGL (DLBCL)	Primary tumor	3 weeks	H2-receptor antagonist
	66	Strauchen et al (1987)	84/M	Pyloric antrum	PGL (DLBCL)	Primary tumor	1 month	H2-receptor antagonist
	67	Harvey et al (1988)	78/F	Gastric body/fundus	GEP-NET (ECL-cell Carcinoid)	Multifocal gastric lesions (majority)	10 years	Not reported
	68	Harvey et al (1988)	55/M	Stomach	GEP-NET (ECL-cell Carcinoid)	Multifocal gastric lesions	5 years	Not reported
	69	Sawant et al (1989)	40/F	Unspecified stomach	GEP-NET (Carcinoid)	Primary tumor	1 year	Biopsy
	70	Shigematsu et al (1989)	40/F	Pyloric antrum	PGL (DLBCL)	Primary tumor (partial)	2 months	Not reported
	71	Shigematsu et al (1989)	73/M	Pyloric antrum	PGL (DLBCL)	Primary tumor	2 months	Not reported
	72	Rebollo et al (1990)	77/M	Gastric body/fundus	GAC	Primary tumor	8 months	Infection (abdominal wall abscess)
	73	Yoshimine et al (1991)	69/F	Gastric angulus	PGL	Primary tumor	2.5 months	Dislodged (ulceration)
	74	Hayakawa et al (1992)	62/F	Lesser curvature	PGL (DLBCL)	Primary tumor	1 year	Necrosis & Detachment
	75	Takehara et al (1992)	44/M	Gastric angulus/Antrum	PGL	Primary tumor (partial)	1 month	H2-blocker
	76	Matsusaki et al (1996)	64/F	Pyloric antrum	PGL (DLBCL)	Primary tumor	1 month	Not reported
	77	Ogawa et al (1998)	63/F	Gastric corpus	PGL (DLBCL)	Primary tumor	13 months	H Pylori eradication
	78	Bariol et al (2001)	24/M	Gastric antrum	PGL (TCL)	Primary tumor	2 years	H Pylori eradication
	79	Salam et al (2001)	73/F	Greater curvature	PGL (DLBCL)	Primary tumor	2.5 years	H Pylori eradication
	80	Pentimone et al (2002)	84/M	Residual stomach	PGL (MALT)	Recurrences	15/5 years	Not reported
	81	Chung et al (2003)	48/M	Lesser curvature	GAC	Primary tumor	4 years	Ischemia (angiography)
	82	Watanabe et al (2003)	22/M	Gastric body & Antrum	PGL (TCL)	Primary tumor	1 month	Infection (Severe EBV viremia in CAEBV)
	83	Watari et al (2005)	60/F	Gastric angulus/Antrum	PGL (DLBCL)	Primary tumor	1 year	H2-blocker; H. pylori eradication
	84	Watari et al (2005)	61/M	Gastric angulus	PGL (DLBCL)	Primary tumor	6 months	H2-blocker; H. pylori eradication
	85	Ohno et al (2006)	14/M	Lower gastric corpus	PGL (MALT)	Primary tumor	10 years	Immunological (Cessation of exposure to H pylori antigen)
	86	Lee et al (2010)	84/M	Cardia & Lower body	GAC	Primary tumor	1 year	Not reported
	87	Ip et al (2011)	77/M	Gastric cardia	GEP-NET (LCNEC)	Primary tumor	3 months	Infection (cytomegalovirus); Cross-autoimmune reaction against neuronal cells
	88	Yang et al (2012)	77/M	Gastric body	GAC	Primary tumor & recurrences	Multiple	Not reported
	89	Rojas-Hernandez et al (2014)	57/M	Greater curvature	PGL (DLBCL)	Primary tumor	2 years	Immunological (B-cell stimulation by HCV)
	90	Shibata et al (2016)	75/M	Gastric antrum	GEP-NET	Peripancreatic lymph metastases	6 months*	EUS-FNA; Bacterial infection
	91	Sugiyama et al (2018)	62/F	Gastric body	PGL (DLBCL)	Primary tumor	10 years	Not reported
	92	Bonilla et al (2019)	78/F	Gastric antrum	GAC	Retroperitoneal adenopathies	3 months	Abscopal effect
	93	Hatsuse et al (2019)	82/F	Unspecified stomach	PGL (DLBCL)	Primary tumor	2 years	Not reported
	94	Okamoto et al (2021)	37/M	Gastric antrum	GEP-NET (Gastrinoma)	Primary tumor	3 years	Biopsy; Resection of omental metastases
	95	Zafar et al (2021)	74/M	Lesser curvature	GAC	Primary tumor	6 years	Not reported
Primary peritoneal cancer	96	Schwartz et al (1991)	39/M	Peritoneum, omentum	MPM	Local regression	8 years	Fever; Rheumatoid factor
	97	BaniHani et al (2009)	38/M	Mesentery	ASPS	Abdominal mass, heart & lung metastases	5 months	Immunological; Herbal medicine
	98	Jagodic et al (2018)	66/F	Retroperitoneal space	RLMS	Liver metastases	2 years	Not reported (possible delayed response to ChT)
Hepatobiliary Cancer	99	Gottfried et al (1982)	65/M	Diffuse hepatic	HCC	Primary tumor	4 years	Abstinence from alcohol; A-P shunt; Portal vein thrombosis
	100	Lam et al (1982)	50/M	Unspecified liver	HCC	Primary tumor & lung metastases	13 years	Chinese herbal medicine; Bronchopneumonia
	101	McCaughan et al (1985)	28/M	Right lobe	HCC	Primary tumor	6.5 years	Androgen withdrawal
	102	McCaughan et al (1985)	40/M	Right lobe	HCC	Primary tumor	9 years	Androgen withdrawal
	103	Sato et al (1985)	78/M	Right lobe	HCC	Primary tumor & bone metastases	5 years	Ischemia (GI Bleeding)
	104	Takayasu et al (1986)	38/M	Unspecified liver	HCC	Primary tumor (partial)	2 months	Subintintimal injury (angiography)
	105	Takayasu et al (1986)	58/F	Unspecified liver	HCC	Primary tumor	2.5 years	Subintintimal injury (angiography)
	106	Andreola et al (1987)	75/M	S6/7	HCC	Primary tumor	18 days	Venous thrombosis
	107	Saez-Royeula (1989)	66/M	Unspecified liver	HCC	Primary tumor	2.5 years	Not reported
	108	Suzuki et al (1989)	65/M	Posterior right lobe	HCC	Primary tumor	6 years	Rapid growth
	109	Ayres et al (1990)	63/F	Diffuse hepatic	HCC	Primary tumor (partial)	1 year	Not reported
	110	Gaffey & Joyce (1990)	63/M	Right lobe	HCC	Primary tumor (partial)	1.5 years	Ischemia (GI Bleeding); Macrobiotic diet
	111	Tocci, G et al (1990)	79/M	Hepatic hilum	HCC	Primary tumor	3 months	Ischemia (GI Bleeding)
	112	Mochizuki et al (1991)	61/M	Unspecified liver	HCC	Primary tumor (partial)	1.5 years	Abscopal Effect
	113	Yamamoto et al (1991)	58/M	Unspecified liver	HCC	Primary tumor	Not reported	Ischemia (hemorrhage)
	114	Yamamoto et al (1991)	68/F	Unspecified liver	HCC	Primary tumor	Not reported	Not reported
	115	Chien et al (1992)	65/M	Right lobe	HCC	Primary tumor	2.5 years	Herbal Remedies
	116	Imaoka et al (1994)	65/M	Left lateral lobe	HCC	Primary tumor	Not reported	Arterial thrombosis
	117	McDermott & Khettry (1994)	23/F	Left lobe	Clear cell HCC	Primary tumor (partial)	5 years	Not reported
	118	Grossmann et al (1995)	52/M	Diffuse hepatic	HCC	Primary tumor (partial)	1 year	Abstinence from alcohol
	119	Herrera et al (1996)	76/M	Unspecified liver	HCC	Primary tumor	1 year	Not reported
	120	Ozeki et al (1996)	69/F	S3	HCC	Primary tumor	1 year	Herbal Remedies
	121	Markovic et al (1996)	62/M	S5/6	HCC	Primary tumor	8 years	Fever; Ischemia (hemorrhage S/P biopsy); Biological effects by cytokines
	122	Yoshimitsu et al (1996)	34/M	Intrahepatic (left lobe)	CCA	Primary tumor	4 months*	Fibrous component
	123	Iwasaki et al (1997)	72/F	Posterior/lateral	HCC	Primary tumor (partial)	1.5 years	Tumor's rapid growth
	124	Van Halteren et al (1997)	72/F	Right lobe	HCC	Primary tumor	2 years	Ischemia & infarction due to Cirrhosis
	125	Kaczynski et al (1998)	73/M	Central part/Hilum	HCC	Primary tumor	3 years	Not reported
	126	Ohba et al (1998)	76/M	S5	HCC	Primary tumor (partial)	2 years	Abscopal Effect
	127	Magalotli et al (1998)	66/M	Unspecified liver	HCC	Primary tumor	4 years*	Not reported
	128	Megalotli et al (1998)	75/F	Unspecified liver	HCC	Primary tumor (partial)	3 years*	Not reported
	129	Sanz et al (1998)	66/M	Right lobe	HCC	Primary tumor	1 year	Immunological
	130	Stoelben et al (1998)	56/M	S6	HCC	Primary tumor	2 years	Immunological (Resection of tumor); Infection (abscess)
	131	Stoelben et al (1998)	74/M	S6	HCC	Primary tumor	3.5 years	Immunological (resection of tumor); Infection (abscess)
	132	Takeuchi et al (1998)	53/M	S8	HCC	Primary tumor	Not reported	Ischemia (thrombus)
	133	Itoh et al (1999)	58/M	S5	HCC	Primary tumor	13 days	Tumor Hypoxia (Thick capsule)
	134	Toyoda et al (1999)	82/M	Right lobe	HCC	Primary regression (Primary & Lung metastases)	1.5 years	Transition from necrosis to fibrosis
	135	Izuishi et al (2000)	50/M	S2/3	HCC	Primary tumor	5 years	Ischemia; Immunological; Angiography
	136	Jang et al (2000)	54/F	Right lobe	HCC	Primary tumor	4 years	Not reported
	137	Lee et al (2000)	44/M	S4/8	HCC	Partial regression (Primary)	1 year*	Infection; Abstinence from alcohol
	138	Lee et al (2000)	63/M	Right lobe	HCC	Partial regression (Primary)	3 years	Infection; Arterial thrombosis/intimal injury (angiography)
	139	Takeda et al (2000)	68/M	S4/5/6/7/8	HCC	Primary tumor	1 year	Herbal Remedies
	140	Terasaki et al (2000)	72/F	S5	HCC	Primary tumor, peritoneal & splenic metastases	2 years	Apoptosis
	141	Uenishi et al (2000)	65/M	Right lobe	HCC	Primary tumor (partial)	1 year	Abstinence from alcohol; A-P shunt; Portal vein thrombosis
	142	Ikeda et al (2001)	75/M	S7	HCC	Primary tumor (partial)	6 years	Not reported
	143	Jung et al (2001)	58/M	Right lobe	HCC	Primary tumor (partial) & lung metastases	1.5 years*	Herbal Remedies; cessation of smoking;
	144	Kawai et al (2001)	58/M	S6	HCC	Primary tumor	1 month	Ischemia; Immunological; Angiography
	145	Matsuo et al (2001)	72/M	S5	HCC	Primary tumor (partial)	1 year	Immunological; Hypoxia; Inflammatory cell infiltration
	146	Nakai et al (2001)	76/M	Residual liver	HCC	Primary tumor	2 years	Immunological (NK cell response)
	147	Sakurai et al (2001)	65/M	Gallbladder fundus	GBAC	Primary tumor	Not reported	Ischemia; Inflammation; Pancreaticobiliary maljunction
	148	Serrano et al (2001)	71/M	Left lobe	HCC	Primary tumor	3 years	Growth factors; Ischemia (hepatic artery)
	149	Abiru et al (2002)	70/M	Unspecified liver	HCC	Primary tumor, lung & bone metastases	2 years	Immunological (IL-18)
	150	Abiru et al (2002)	65/F	Unspecified liver	HCC	Primary tumor, lung & lymph metastases	4 months	Immunological (IL-18)
	151	Abiru et al (2002)	65/M	Unspecified liver	HCC	Primary tumor, lung & bone metastases	1 year	Immunological (IL-18)
	152	Lee et al (2002)	70/M	S2/3	HCC	Primary tumor	24 days	Occlusion of feeding artery
	153	Misawa et al (2002)	62/M	Anterior segment	HCC	Primary tumor	1 year	Biological effects by A-P shunt
	154	Morimoto et al (2002)	73/M	S2/3	HCC	Primary tumor	1 year	Arterial thrombosis
	155	Zimmermann et al (2002)	56/M	S6	Medullary-like HCC	Primary tumor	2 years	Immunological (Cytotoxic pathway); Apoptosis
	156	Iiai et al (2003)	69/M	S6/7	HCC	Primary tumor	4 years	Portal vein thrombosis; cessation of smoking
	157	Jozuka et al (2003)	52/M	Hepatic surface	HCC	Primary tumor	2.5 years	Psychoneurological; Antidepressants; Immunological
	158	Li et al (2003)	53/M	S6	HCC	Primary tumor	Not reported	Biological effects by cytokines
	159	Ohta et al (2003)	74/M	S2/3	HCC	Primary tumor	1 year	Immunological; hypoxia (arterial sclerosis)
	160	Blondon et al (2004)	64/M	Diffuse hepatic	HCC	Local regression	9 months	Infection (peritonitis); Ischemia (Intraperitoneal bleed)
	161	Blondon et al (2004)	70/F	Diffuse hepatic	HCC	Local regression	3 years	Infection (peritonitis); Ischemia (Intraperitoneal bleed); tamoxifen
	162	Cheng et al (2004)	74/M	Medial left lobe	HCC	Primary tumor	6 years	Herbal remedies
	163	Erturk et al (2004)	69/M	Left lobe	HCC	Primary tumor	3 years	Blood transfusion
	164	Feo et al (2004)	71/F	S3/5	HCC	Primary tumor (partial)	1.5 years	Ischemia
	165	Kato et al (2004)	72/M	Right lobe	HCC	Primary tumor (partial)	2 years	Not reported
	166	Kato et al (2004)	77/M	Right lobe	HCC	Primary tumor & lung metastases	1 year	Abstinence from smoking
	167	Lin et al (2004)	42/M	S8	HCC	Primary tumor (partial)	2 years	Herbal remedies
	168	Nakajima et al (2004)	80/M	S4/6	HCC	Partial regression (Primary)	6 months	Ischemia; Intratumoral bleeding/hemorrhagic necrosis
	169	Jeon et al (2005)	72/M	Right lobe	Clear Cell HCC	Primary tumor (partial) & chest wall metastases	9 months	Metabolic derangement (hypoglycemia & HLD)
	170	Moon et al (2005)	72/M	S6	HCC	Primary tumor	2 years	Alcohol cessation
	171	Nam et al (2005)	65/M	Right lobe	HCC	Liver & bone metastases (partial)	1 year	Abscopal Effect
	172	Nouso et al (2005)	85/M	S5/6/7/8	HCC	Primary tumor (partial)	2 years	Ischemia; Vitamin K administration
	173	Ohtani et al (2005)	69/M	S4	HCC	Primary tumor (partial)	3 years	Tumor Hypoxia (Thick capsule)
	174	Randolph et al (2005)	56/M	Left lateral lobe	HCC	Primary tumor	1.5 years	Alcohol cessation, ischemia (obstruction of portal vein thrombosis); Infection (Pneumonia)
	175	Rizell et al (2005)	58/M	Central liver	HCC	Primary tumor (partial)	1.5 years	Sirolimus (Immunosuppressive)
	176	Yano et al (2005)	71/F	S8	HCC	Primary tumor (partial)	2 years	Hypoxia (artery rupture)
	177	Otrock et al (2006)	75/F	Diffuse hepatic	HEHE	Primary tumor	3.5 years	Not reported
	178	Kojima et al (2006)	79/M	S8	HCC	Lung metastases	6 months	Steroids, Hormones, or Herbal Remedies
	179	Kondo et al (2006)	67/M	S4	HCC	Primary tumor (partial)	2 months	Immunological
	180	Kondo et al (2006)	67/M	S5/3	HCC	Primary tumor & lung metastases	4 years	CAM's; Immunological
	181	Kondo et al (2006)	70/M	Right lobe	HCC	Primary tumor (partial)	5 years	Bleeding (esophageal varicies); Immunological
	182	Kondo et al (2006)	75/M	S7	HCC	Lung metastases (partial)	2 years	Immunological
	183	Shibuya et al (2006)	71/M	S5	HCC	Primary tumor	2 months	Ischemia; Immunological; Angiography
	184	Heianna et al (2007)	70/F	Unspecified liver	HCC	Lung metastases	5 years	Immunological (Host cytokines); Systemic inflammatory (TACE of primary)
	185	Matsunaga et al (2007)	71/F	Left lateral lobe	Sarcomatoid HCC	Peritoneal metastases	4 months*	Ischemia (rapid growth)
	186	Meza-Junco et al (2007)	56/F	S5	HCC	Primary tumor	2 years	Hypoxia (Thick capsule)
	187	Peddu et al (2007)	57/M	S4	HCC	Primary tumor	2 months	Auto-infarction
	188	Vardhana et al (2007)	?/M	S2/3/4	HCC	Primary tumor	8 months	Immunological
	189	Arakawa et al (2008)	78/F	S2/3	HCC	Primary tumor	2.5 years	Immunological; Portal vein thrombosis
	190	Hori et al (2008)	71/M	Gallbladder	GBAC	Primary tumor	Not reported	Increased intraluminal pressure (PBM); Pancreatic enzymes
	191	Sibartie et al (2008)	76/M	S5	HCC	Primary tumor (partial)	2 years	Ischemia (disturbance of blood flow)
	192	Del Poggio et al (2009)	77/F	S6	HCC	Primary tumor (partial)	1.5 years	Immunological (tumor antigens)
	193	Hsu et al (2009)	66/M	S7/8	HCC	Primary tumor (partial)	1.5 years	Hypoxia; Immunological; Silymarin; Portal vein thrombosis
	194	Kanzaki et al (2009)	52/M	S8	HCC	Primary tumor	8 months	Tumor Hypoxia (thick capsule)
	195	Nishijima et al (2009)	86/F	S7	HCC	Primary tumor (partial)	4 months	Tumor infarction
	196	Oquiñena et al (2009)	54/M	S6	HCC	Primary tumor	2 years	Vascular ischemia; Immunological
	197	Oquiñena et al (2009)	61/M	S1	HCC	Primary tumor	1.5 years	Vascular ischemia; Immunological
	198	Oquiñena et al (2009)	60/M	Right lobe	HCC	Primary tumor	3 years	Vascular ischemia; Immunological
	199	Park et al (2009)	57/M	S5/6/7/8	HCC	Primary tumor (partial)	5 years	Infiltrating lymphocytes
	200	Harada et al (2010)	70/M	S7	HCC	Primary tumor	2 years	Ischemia; Herbal remedies
	201	Hong et al (2010)	67/M	Resection margin	HCC	Primary tumor & lung metastases	1 year	TACE of Primary
	202	Kai et al (2010)	58/F	S6/7	HCC	Primary tumor	1 month	intimal injury (angiography)
	203	Kai et al (2010)	49/M	S6	HCC	Primary tumor	3 weeks	intimal injury (angiography)
	204	Satou et al (2010)	83/M	Right lobe	HCC	Primary tumor	Not reported	NSAIDS (Ketoprofen)
	205	Storey et al (2010)	52/M	S5/6	HCC	Primary tumor	3 years	Cessation of alcohol
	206	Alqutub et al (2011)	65/M	Right lobe	HCC	Primary tumor	2 years	Ischemia (Rapid growth; Intratumoral hemorrhage)
	207	Arora et al (2011)	54/M	Right lobe	HCC	Primary tumor	2 years	Immunological; Necrosis
	208	Fukushima et al (2011)	69/M	Right lobe	HCC	Lung metastases	7 years	Immunological (TACE of Primary)
	209	Maejima et al (2011)	68/M	S3/5	HCC	Primary tumor	3 months	Ischemia; Immunological
	210	Okano et al (2011)	68/M	Right lobe	HCC	Tumor recurrence	2 years	PVT; Ischemia (rapid growth)
	211	Okuma et al (2011)	63/M	Right lobe	HCC	Lung metastases	3 years	Abscopal Effect
	212	Bastawrous et al (2012)	63/M	Right lobe	HCC	Primary tumor (partial)	Not reported	Ischemia
	213	Harimoto et al (2012)	73/M	S6/7	HCC	Primary tumor & lung metastases	1 year	Ischemia (hypotension during dialysis)
	214	Komatzu et al (2012)	65/M	Right lobe	HCC	Primary tumor & recurrences	6 months (x3)	Not reported
	215	Nakayama et al (2012)	92/F	Right lobe	HCC	Primary tumor	Not reported	Ischemia (rapid growth); Immunological
	216	Takeura et al (2012)	69/F	Unspecified liver	HCC	Primary tumor & bone metastases	10 months	Inflammatory (trauma)
	217	Takeura et al (2012)	84/F	Unspecified liver	HCC	Primary tumor & peritoneal carcinomatosis	1.5 years	Inflammatory (trauma)
	218	Yamamoto et al (2012)	60/M	S4-8	HCC	Primary tumor	3 weeks	Immunological; Diabetes Control
	219	Yokoyama et al (2012)	80/M	S4	HCC	Primary tumor	1 month	Immune; Ischemia (thrombus)
	220	Katayama et al (2013)	74/M	S5/6	HCC	Primary tumor	1 month	Tumor Hypoxia (thick capsule)
	221	Okano et al (2013)	77/M	S4/6/7/8	HCC	Primary tumor (partial)	1 year	Ischemia (Disruption of feeding artery); Abstinence from alcohol
	222	Sasaki et al (2013)	79/M	S2	HCC	Primary tumor	2 months	Not reported
	223	Tomishige et al (2013)	76/F	S6	HCC	Primary tumor	Not reported	Not reported
	224	Ueda et al (2013)	63/F	S7	HCC	Primary tumor	Not reported	Ischemia (Hypotension during dialysis)
	225	Bhardwaj et al (2014)	74/M	Left lobe	HCC	Primary tumor	Not reported	Not reported
	226	Chiesara et al (2014)	65/M	S6	HCC	Primary tumor (partial)	2 years	Herbal remedies; Ischemia; Inflammatory Processes
	227	Inoue et al (2014)	57/M	S5	HCC	Primary tumor	Not reported	Drugs (Inhibition of angiogenesis by rifampicin & minocycline)
	228	Lim et al (2014)	64/M	Right lobe	HCC	Primary tumor	6 months	Immunological; Herbal medicine
	229	Miyake et al (2014)	79/M	S6/8	HCC	Primary tumor	1 month	Tumor Hypoxia (thick capsule)
	230	Saito et al (2014)	74/M	S8	HCC	Primary tumor (partial)	2 months	Immunological; Cessation of drinking & smoking
	231	Tomino et al (2014)	77/M	S1	HCC	Primary tumor	1 month	Hypoxia; Fever; Biopsy
	232	Tsai et al (2014)	74/M	Left lobe	HCC	Primary tumor	4 years	Not reported
	233	Zhao et al (2014)	22/F	Diffuse hepatic	HEHE	Primary tumor (partial)	3 years	Not reported
	234	Parks et al (2015)	69/M	S8	HCC	Recurrent Hepatic Lesions	6 months*	Immunological (Vitiligo autoimmunity)
	235	Parks et al (2015)	63/M	S7	cHCC-CC	Retroperitoneal lymph metastases	2 months	Immunological
	236	Parks et al (2015)	67/M	Left lobe	HCC	Primary tumor	5 months	Immunological
	237	Kim et al (2015)	57/M	S6	HCC	Primary tumor	Not reported	Immunological; Ischemia
	238	Kohda et al (2015)	80/M	S1	HCC	Primary tumor	Not reported	Ischemia (rapid growth)
	239	Kuwano et al (2015)	84/M	S4	HCC	Primary tumor	Not reported	Ischemia
	240	Matsuoka et al (2015)	67/M	S6	HCC	Primary tumor	3 years	Hypoxia; Hepatic arterial & portal vein thromboses
	241	Okano et al (2015)	73/M	S8	HCC	Primary tumor	6 months	Ischemia; Angiography
	242	Sugamoto et al (2015)	77/F	S3	HCC	Primary tumor	9 months	Immunological (weight loss)
	243	Takeda et al (2015)	68/M	S4	HCC	Primary tumor (partial)	1 year	Hypoxia; Vessel thrombosis
	244	Tazawa et al (2015)	77/M	S7	HCC	Primary tumor	3 months	Ischemia (postoperative hypotension)
	245	Verla-Tebit et al (2015)	53/M	Right lobe	HCC	Primary tumor & lung metastases (partial)	1.5 years	Anti-hepaciviral medication for Hepatitis C (sorafenib & ribavirin)
	246	Wang et al (2015)	50/M	S7/8	HCC	Primary tumor	Not reported	Immunological
	247	Yang et al (2015)	59/M	S6	HCC	Primary tumor	6 months	Seroconversion of HBV
	248	Yoo et al (2015)	62/M	S5	HCC	Primary tumor (partial)	2 years	Immunological; Hypoxia/Ischemia
	249	Gunasekaran et al (2016)	49/M	Left lobe	HCC	Pulmonary metastases	5 months	Consumption of Guaynabo fruit extract
	250	Heron et al (2016)	61/F	S7	HCC	Primary tumor	1 year	Withdrawal of azathioprine in Crohn's Disease; Biopsy
	251	Jianxin et al (2016)	64/M	S6	HCC	Tumor recurrence and omental metastases	2.5 years	Immunological; Herbal medicine
	252	Kumar et al (2016)	40/M	S2/3/5	HCC	Primary tumor	7 years	Cessation of immunosuppressive therapy
	253	Kumar et al (2016)	74/M	Right	HCC	Primary tumor (partial)	8 months	Cessation of immunosuppressive therapy
	254	Luo et al (2016)	61/F	S4	HCC	Primary tumor	2.5 years	Cirrhosis related hypoxia
	255	Mahmood et al (2016)	59/M	S4/8	HCC	Primary tumor	4 months	Anti-hepaciviral medication for Hepatitis C (sorafenib & ribavirin)
	256	Ooka et al (2016)	63/M	S7/8	HCC	Primary tumor	6 months	Ischemia (PVT)
	257	Pectasides et al (2016)	53/M	S4	HCC	Primary (partial) & lung metastases (partial)	2 months	Portal vein thrombosis; Immunological reaction
	258	Sawatsubashi et al (2016)	59/M	S5-8	HCC	Primary tumor	1 month	Tumor Hypoxia (thick capsule)
	259	Sugiura et al (2016)	90/F	S6	HCC	Primary tumor	Not reported	Not reported
	260	Alam et al (2017)	65/M	S5/6	HCC	Primary tumor (partial)	3 months	Immunological
	261	Iwatani et al (2017)	59/M	S8	HCC	Primary tumor	Not reported	Ischemia (Duodenal Ulcer)
	262	Murata et al (2017)	67/M	S1/8	HCC	Primary tumor	2 months	Ischemia
	263	Noij et al (2017)	74/M	Diffuse hepatic	HCC	Primary (partial) & lung metastases	6 months	Not reported
	264	Oyama et al (2017)	79/M	Diffuse hepatic	HCC	Primary tumor	Not reported	Hypoxia
	265	Oyama et al (2017)	78/F	Left lobe	HCC	Primary tumor	Not reported	Inflammatory; Immunological; Infection (Bacterial)
	266	Sakamaki et al (2017)	78/M	S8	HCC	Primary tumor & lymph metastases	3 months	Immunological; hemodialysis
	267	Sano et al (2017)	30/F	Common bile duct	NET	Primary tumor	Not Reported	Biopsy; Central necrosis
	268	Yamaguchi et al (2017)	63/M	Right lobe	HCC	Primary tumor	Not reported	Not reported
	269	Yamaguchi et al (2017)	67/M	S5	HCC	Primary tumor	Not reported	Not reported
	270	Yamaguchi et al (2017)	84/F	S7	HCC	Primary tumor	Not reported	Not reported
	271	Yamaguchi et al (2017)	60/M	S8/S1	HCC	Primary tumor	Not reported	Not reported
	272	El-Badrawy et al (2018)	45/F	Porta hepatis	DLBCL	Primary tumor	18 days	Biopsy (Aspiration); Regional immune reaction
	273	Goto et al (2018)	64/M	S6/7	HCC	Primary tumor	1 month	Portal vein thrombosis; Immunological
	274	Koya et al (2018)	83/M	S2/3/4	HCC	Primary tumor	1 year	PVT
	275	Lee et al (2018)	67/M	Diffuse hepatic	HCC	Primary tumor	1 year	Infection (diabetic foot); Ischemia (obstruction of portal vein thrombosis)
	276	Taniai et al (2018)	74/M	S7	HCC	Primary tumor	2 years	Not Reported
	277	Taniguchi et al (2018)	70/M	S3	HCC	Primary tumor	Not reported	Ischemia (dialysis); Drugs (Elythrocin Steroids)
	278	Alhatem et al (2019)	60/M	Right lobe	DLBCL	Primary tumor	4 years	Immunological (HIV, Hep C); Genetic
	279	Chohan et al (2019)	79/F	S6	HCC	Primary (partial) & lung metastases	1.5 years*	Ischemia; Immunological
	280	Fujikawa et al (2019)	78/M	S2/7	HCC	Primary tumor	Not reported	Anemia (fracture)
	281	Hirota et al (2019)	67/M	S7	HCC	Primary tumor	Not reported	Ischemia; Cessation of alcohol and smoking
	282	Kim et al (2019)	70/M	Bile duct	CCA	Liver Metastasis	3 months	Abscopal effect; Post-radiotherapy antitumoral immunity
	283	Kawaguchi et al (2019)	68/M	S3	HCC	Primary tumor	2.5 months	Antiangiogenesis (SGLT2i)
	284	Lee et al (2019)	78/F	S5-8	HCC	Primary tumor	1 month	Immunological; Ischemia (rapid tumor growth or disruption of feeding artery)
	285	Yoshida et al (2019)	71/F	Right lobe	Clear cell HCC	Primary tumor	1 year	Not reported
	286	Arjunan et al (2020)	53/M	Liver	HCC	Pulmonary, Omental, retroperitoneal metastases	5 years	Immunological
	287	Arjunan et al (2020)	48/M	Left Lobe	HCC	Pulmonary metastases	13 years	Immunological
	288	Arjunan et al (2020)	62/M	Liver	HCC	Systemic metastases	11 years	Immunological
	289	Arjunan et al (2020)	73/M	Liver	HCC	Primary tumor	6 years	Immunological
	290	Costa-Santos et al (2020)	68/M	S4	HCC	Hepatic lesions	5 years	Megestrol; Herbal remedies
	291	Hokkoku et al (2020)	77/M	S6	HCC	Primary tumor	Not reported	Not reported
	292	Muroya et al (2020)	78/M	Right lobe	HCC	Lung metastases	1 year	Hypoxia; Immunological; Dialysis
	293	Nakamoto et al (2020)	74/M	Right lobe	HSTCL	Hepatic, splenic, & osseous lesions	1.5 months	Biopsy
	294	Ohmatsu et al (2020)	77/M	Right lobe	HCC	Lung metastases	1 month	Abscopal Effect
	295	Onishi et al (2020)	28/M	Left lobe	HEHE	Primary lesion (partial)	6 years*	Unpredictable growth (new lesions)
	296	Onishi et al (2020)	44/M	Unspecified liver	HEHE	Primary lesion (partial)	4 years*	Unpredictable growth (new lesions)
	297	Onishi et al (2020)	47/M	Unspecified liver	HEHE	Primary lesion (partial)	12 years	Calcification
	298	Onishi et al (2020)	51/F	S6	HEHE	Primary lesion (partial)	11.5 years*	Unpredictable growth (new lesions)
	299	Onishi et al (2020)	61/F	Unspecified liver	HEHE	Primary lesion (partial)	5.5 years*	Unpredictable growth (new lesions)
	300	Onishi et al (2020)	63/M	Unspecified liver	HEHE	Primary lesion (partial)	6 years*	Unpredictable growth (new lesions)
	301	Raufi et al (2020)	63/M	Porta hepatis	PHNEC	Pulmonary metastases	2 months	Immunological
	302	Sakamoto et al (2020)	62/M	S3	HCC	Primary tumor	Not reported	Ischemia
	303	Sakamoto et al (2020)	75/F	S4	HCC	Primary tumor	Not reported	Tumor hypoxia (bleeding from rectal varicose veins)
	304	Sonbare et al (2020)	74/M	S8	HCC	Primary tumor	1 year	Immunological; Ischemia
	305	Franses et al (2021)	64/M	S4	HCC	Primary tumor	2 months	Immunological
	306	Kakuta et al (2021)	71/M	Not reported	HCC	Lung metastases	3 months*	TACE of Primary
	307	Kimura et al (2021)	84/F	S8	HCC	Primary tumor (partial)	Not reported	Immunological
	308	Liu et al (2021)	67/M	Diffuse hepatic	HCC	Pulmonary metastases	5 months	Immunological; Chinese herbal remedies
	309	Obu et al (2021)	83/M	S2	HCC	Primary tumor	1 year	Ischemia (rapid growth); Capsule formation; PVT
	310	Tanaka et al (2021)	71/F	Diffuse hepatic	HHL	Hepatic lesions	1 year	Cessation of immunosuppressive therapy
Pancreatic cancer	311	Shapiro (1967)	?/F	Unspecified pancreas	PDAC	Primary tumor	7.5 years	Not reported
	312	Lokich et al (1973)	42/M	Pancreatic head	PDAC	Primary tumor	2 years*	Not reported
	313	Eidemiller et al (1971)	?/M	Pancreatic head	PDAC	Primary tumor	6 years	Not reported
	314	Tchertkoff et al (1974)	21/M	Pancreatic head	PDAC	Primary tumor	12 years	Bacterial Infection
	315	Cann et al (2003)	50/M	Pancreatic body	PDAC	Primary tumor	6 months	Acute febrile response; alternative therapies; Chinese herbs; High-dose Vitamin C
	316	Chin et al (2017)	77/M	Pancreatic head	PDAC	Primary tumor & liver metastases	1 year	Leukocyte activation; Fever; Allergenic & hormonal influences
	317	Sreevathsa et al (2018)	32/M	Pancreatic body	pNET (Carcinoid)	Primary tumor	19 years	Apoptosis (cytokines); VEGF blockade
	318	Lemus et al (2019)	56/F	Pancreatic body/tail	PDAC	Primary tumor & liver metastases	3 years	Immunogenic; angiogenic effects on the tumor microenvironment.
	319	Ibrahimi et al (2019)	59/F	Residual pancreas	PDAC	Primary tumor (partial)	1 year	Acute pancreatitis; Bacterial/fungal infection (abscess)
	320	Kawaguchi et al (2021)	66/F	Pancreatic tail	PDAC	Primary tumor (partial)	1 month	Not reported
Small bowel cancer	321	Sroujieh et al (1988)	55/M	Occult (Ileal lesion)	MM	Intestinal lesion (occult primary)	7 years	Not Reported
	322	Nagashima et al (1996)	58/M	Duodenum	MALT Lymphoma	Primary tumor	1 year	Eradication of H. Pylori
	323	Rayson et al (1996)	45/F	Ileum	GEP-NET (Carcinoid)	Liver metastases	5 months*	Valvular surgery for carcinoid heart disease
	324	Horiuhi et al (2003)	74/F	Diffuse enteric	NKTCL	Upper Abdominal tumors (non-radiated)	1 year	Abscopal Effect
	325	Makino et al (2010)	38/M	Terminal Ileum	MALT Lymphoma	Primary tumor & ileocecal lymphadenopathy	1 year	Resolution of infection; Inflammatory
	326	Hayashi et al (2013)	64/F	Duodenum	FL	Primary tumor	5.5 years	Eradication of H. Pylori
	327	Tanaka et al (2014)	61/F	Duodenum	SBAC	Primary tumor & liver metastases	4 months	Methotrexate
	328	Sasaki et al (2016)	60/M	Ileum	RL	Primary tumor	Not reported*	Radiography Radiation
	329	Hori et al (2017)	20/F	Small intestine	EAS	Lung & mediastinal lymph metastases	2 months	Immunological; Biopsy (transbronchial); Inflammatory
	330	Tanaka et al (2019)	35/M	Small intestine	DLBCL	Primary tumor & lymph metastases	3 years	Immunological (PD-L1/PD-1 axis)
Colorectal cancer	331	Most (1927)	57/M	Rectum	CRAC	Local recurrence	9 years	Sepsis
	332	Henry (1944)	60/M	Rectum	CRAC	Primary tumor & liver metastases	11 years	Not reported
	333	Fergeson (1954)	45/M	Descending colon	CRAC	Primary tumor with local extension	10 years	Severe sepsis (abscess)
	334	Dunphy (1956)	46/M	Rectum	CRAC	Primary tumor & liver metastases	8 years	Severe debilitation; Fecal diversion
	335	Ellison (1956)	59/M	Rectum	CRAC	Peritoneal carcinomatosis	3 years	Persistent high fever (Pneumonia)
	336	Fallis (1959)	42/M	Transverse colon	CRAC	Primary tumor with local extension	18 years	Severe sepsis (abscess); Fecal diversion; Religious rituals
	337	Brown (1961)	54/F	Sigmoid colon	CRAC	Primary tumor & liver metastases	3 years	Not reported
	338	Brunschwig et al (1963)	68/F	Rectum	CRAC	Local recurrence	14 years	Not reported
	339	Mayo et al (1963)	63/F	Descending colon	CRAC	Liver metastases	16 years	Not reported
	340	Fullerton & Hill (1963)	58/F	Transverse colon	Anaplastic CRAC	Primary tumor	16 years	Not reported
	341	Rankin et al (1965)	44/M	Rectum	CRAC	Liver metastases	9 years	Not reported
	342	Margolis & West (1967)	71/M	Rectum	CRAC	Peritoneal carcinomatosis	1 year	Fecal diversion
	343	Synder et al (1968)	62/F	Sigmoid colon	CRAC	Primary tumor with local extension	15 years	Persistent high fever (wound infection); Immunologic; Genetic
	344	Synder et al (1968)	60/M	Cecum	CRAC	Peritoneal carcinomatosis	9 years	Severe Sepsis (abscess w/ fecal fistula); Immunologic; Genetic
	345	Weinstock (1977)	40/F	Sigmoid colon	CRAC	Primary tumor with local extension & liver metastases	20 years	Psychological
	346	Meares (1979)	64/M	Rectum	CRAC	Primary tumor	1 year	Intensive meditation
	347	Glasser et al (1979)	36/M	Ascending colon	CRAC	Primary tumor & peritoneal carcinomatosis	28 years	Genetic factors
	348	Beechy et al (1986)	23/F	Ascending colon	CRAC	Primary tumor with local extension, peritoneal, & liver metastases	4 years	Not reported
	349	Tominaga et al (1999)	44/F	Transverse colon	CRAC	Primary tumor	3 years	Dislodged
	350	Wadsworth et al (1999)	67/M	Rectum	CRAC	Pulmonary metastases	3 years	Infection (Pneumonia)
	351	Okamura et al (2000)	54/M	Rectum	MALT Lymphoma	Primary tumor	Not reported	Not reported
	352	Kamesui et al (2000)	66/F	Ascending colon	CRAC	Primary tumor	1 year	Dislodged
	353	Takenaka et al (2000)	76/F	Rectum	MALT Lymphoma	Primary tumor	1.5 years	Not reported
	354	Ikuta et al (2002)	60/M	Rectum	ASC	Liver metastases	2 years	Interruption of blood supply; growth factors
	355	Abdelrazeq et al (2005)	51/M	Rectum	CRAC	Local recurrence & peritoneal carcinomatosis	16 years	Immunologic; Metabolic; Endocrine; Diversion of carcinogen
	356	Itano et al (2006)	63/M	Rectum	MM	Primary tumor	2 years	Dislodged
	357	Tomiki et al (2007)	80/F	Rectum	CRAC	Primary tumor	5 years	Dislodged
	358	Kochi et al (2008)	80/M	Transverse colon	CRAC	Primary tumor	3 years	Dislodged
	359	Bir et al (2009)	86/F	Ascending colon	CRAC	Peritoneal lymph node metastases	2 years	Immunologic
	360	Sakamoto et al (2009)	80/M	Rectum	CRAC	Primary tumor	3 months	Immunological
	361	Shimizu et al (2010)	80/M	Transverse colon	CRAC	Primary tumor	5 years	Physical stimulation (peristaltic movement; dislodged)
	362	Sakuma et al (2011)	64/M	Sigmoid colon	CRAC	Primary tumor	3 months*	Not reported
	363	Nakashima et al (2012)	76/F	Ileocecal junction	CRAC	Primary tumor	2 months	Dislodged
	364	Flynn et al (2013)	36/M	Descending colon/rectum	DLBCL	Primary tumor	6 months	Cessation of immunosuppressive therapy (infliximab & azathioprine)
	365	Flynn et al (2013)	52/M	Sigmoid colon	HL	Primary tumor	1 year	Cessation of immunosuppressive therapy (infliximab & azathioprine)
	366	Nakamura et al (2013)	60/M	Rectum	CRAC	Primary tumor	1.5 years	Biopsy; Immunological
	367	Sekiguchi et al (2013)	76/F	Cecum	CRAC	Primary tumor	1.5 months	Hyperimmunity & perioperative stress (lung surgery)
	368	Kihara et al (2014)	64/M	Transverse colon	CRAC	Primary tumor	1.5 months	Immunological
	369	Mitchell et al (2014)	75/F	Cecum	MC	Regional lymph metastases	1 year	Immunological
	370	Sewpaul et al (2014)	35/F	Appendix (presumed)	GEP-NET (Carcinoid)	Primary tumor	1 year	Pregnancy
	371	Kyoichi et al (2015)	65/M	Transverse colon	CRAC	Primary tumor	1.5 months	Drugs (Metformin); Immunological
	372	Serizawa et al (2015)	75/M	Transverse colon	CRAC	Primary tumor	2.5 months	Immunological; Apoptosis
	373	Ito et al (2016)	73/M	Ascending colon	CRAC	Primary tumor	3.5 months	Biopsy; mechanical stimulation (intestinal peristalsis)
	374	Nemésio et al (2016)	51/F	Splenic angle	CRAC	Liver metastases	Not reported	Removal of Primary; Tumor necrosis
	375	Chida et al (2017)	80/M	Transverse colon	CRAC	Primary tumor	1 month	Immunological (CD4 +T-cells)
	376	Matsuki et al (2016)	72/F	Ascending colon	CRAC	Liver metastases	2 months	Infection (Biliary S/P pancreaticoduodenectomy)
	377	Chuang et al (2018)	74/F	Occult	CRAC	Lung metastases	2 months	Abscopal effect
	378	Yoshida et al (2018)	73/M	Transverse colon	CRAC	Primary tumor	2.5 months	Not reported
	379	Fukutomi et al (2019)	87/F	Transverse colon	CRAC	Primary tumor	1 month	Immunological (CD8+ T-cells)
	380	Karakuchi et al (2019)	70/M	Transverse colon	CRAC	Primary tumor	2 months	Immunological; Biopsy
	381	Kawakita et al (2019)	62/M	Ascending colon	CRAC	Primary tumor	1 month	Immunological (CD4 +T-cells)
	382	Tanaka et al (2019)	63/F	Sigmoid colon	CRAC	Primary tumor	2.5 months	Injection for non-lifting sign; Ischemia (vasoconstriction S/P epinephrine)
	383	Utsumi et al (2020)	78/M	Ascending colon	CRAC	Primary tumor	1 month	Immunological (dMMR)
	384	Utsumi et al (2020)	66/M	Ascending colon	CRAC	Primary tumor	1.5 months	Immunological (dMMR)
	385	Utsumi et al (2020)	73/M	Ascending colon	CRAC	Primary tumor	1.5 years	Immunological (dMMR)
	386	Mehawej et al (2020)	18/F	Occult	CRAC	Primary tumor	Unknown	Not reported
	387	Nishiura et al (2020)	67/F	Transverse colon	CRAC	Primary tumor	3 months	Immunological
	388	Yokota et al (2020)	76/F	Transverse colon	CRAC	Primary tumor	2 months	Immunological
	389	Yokota et al (2020)	64/F	Cecum	CRAC	Primary tumor	3 months	Immunological
	390	Yokota et al (2020)	64/M	Transverse colon	CRAC	Primary tumor	1 month	Immunological

1	Roxburgh, D.: Spontaneous Regression of Cancer. Brit. M. J., 1: 39, 1935
2	Grillet B, Demedts M, Roelens J, Goddeeris P, Fossion E. Spontaneous regression of lung metastases of adenoid cystic carcinoma. Chest. 1984 Feb;85(2):289-91. doi: 10.1378/chest.85.2.289. PMID: 6319089
3	Grillet B, Demedts M, Roelens J, Goddeeris P, Fossion E. Spontaneous regression of lung metastases of adenoid cystic carcinoma. Chest. 1984 Feb;85(2):289-91. doi: 10.1378/chest.85.2.289. PMID: 6319089.
4	Grem, J. L., Hafez, G. R., Brandenburg, J. H. et a/. (1986) Spontaneous remission in diffuse large cell lymphoma. Cancer 57: 2042-2044.
5	Poppema S., Postma L., Brinker M., Jong B. Spontaneous regression of a small non-cleaved-cell malignant lymphoma (non-Burkitt’s lymphoblastic lymphoma): morphologic, immunohistochemical and immunoglobulin gene analysis. Cancer. 1988;62:791–794.
6	Savarrio L, Gibson J, Dunlop DJ, O'Rourke N, Fitzsimons EJ. Spontaneous regression of an anaplastic large cell lymphoma in the oral cavity: first reported case and review of the literature. Oral Oncol. 1999 Nov;35(6):609-13. doi: 10.1016/s1368-8375(99)00034-2. PMID: 10705098.
7	King M, Spooner D, Rowlands DC. Spontaneous regression of metastatic malignant melanoma of the parotid gland and neck lymph nodes: a case report and a review of the literature. Clin Oncol (R Coll Radiol). 2001;13(6):466-9. doi: 10.1053/clon.2001.9315. PMID: 11824888.
8	Notani, Kenichi & Shindoh, Masanobu & Takami, Tsuyoshi & Yamazaki, Yutaka & Kohgo, Takao & Fukuda, Hiroshi. (2002). Anaplastic Large Cell Lymphoma (ALCL) in the Oral Mucosa with Repeating Recurrence and Spontaneous Regression of Ulceration: Report of a case. Oral Medicine & Pathology. 7. 79-82.
9	Koga M, Kusukawa J, Hayabuchi N. Spontaneous regression of extranodal malignant lymphoma occurred in the gingiva. Oral Oncol. 2003 Apr;39(3):323-4. doi: 10.1016/s1368-8375(02)00122-7. PMID: 12618208.
10	Yamamoto, K., Ohgi, K., Yasumoto, J., Imai, Y., Kawakami, M., & Kirita, T. (2003). A case of spontaneous regression of malignant lymphoma of the upper gingiva. In Japanese) J Jpn Soc Oral Tumors, 15, 7-12.
11	Yokoyama T, Miyazawa K, Otawa M, Kawakubo K, Kuriyama Y, Serizawa H, Mukai K, Ohyashiki K. [Spontaneous regression of MALT lymphoma of the hard palate accompanied by Sjögren syndrome]. Rinsho Ketsueki. 2003 Jul;44(7):468-70. Japanese. PMID: 12931566.
12	Heibel H, Knödgen R, Bredenfeld H, Wickenhauser C, Scheer M, Zöller JE. Complete spontaneous remission of an aggressive non-Hodgkin's lymphoma with primary manifestation in the oral cavity. Leuk Lymphoma. 2004 Jan;45(1):171-4. doi: 10.1080/1042819031000139747. PMID: 15061215.
13	Lester R, Li C, Phillips P, Shenkier TN, Gascoyne RD, Galbraith PF, Vickars LM, Leitch HA. Improved outcome of human immunodeficiency virus-associated plasmablastic lymphoma of the oral cavity in the era of highly active antiretroviral therapy: a report of two cases. Leuk Lymphoma. 2004 Sep;45(9):1881-5.
14	Sakuma H, Okabe M, Yokoi M, Eimoto T, Inagaki H. Spontaneous regression of intraoral mucosa-associated lymphoid tissue lymphoma: molecular study of a case. Pathol Int. 2006 Jun;56(6):331-5. doi: 10.1111/j.1440-1827.2006.01967.x. PMID: 16704497.
15	Armstrong R, Bradrick J, Liu YC. Spontaneous regression of an HIV-associated plasmablastic lymphoma in the oral cavity: a case report. J Oral Maxillofac Surg. 2007 Jul;65(7):1361-4. doi: 10.1016/j.joms.2005.12.039. PMID: 17577503.
16	Kurita M, Hirano K, Ebihara S, et al. Spontaneous regression of cervical lymph node metastasis in a patient with mesopharyngeal squamous cell carcinoma of the tongue: possible association between apoptosis and tumor regression. Int J Clin Oncol. 2007; 12(6): 448- 454.
17	Oya, Ikemura K. Spontaneous regression of recurrent squamous cell carcinoma of the tongue. Int J Clin Oncol. 2004 Aug;9(4):339-42. doi: 10.1007/s10147-004-0404-6.
18	Rujirojindakul P, Kayasut K, Rohitoprakarn M, Lekhakula A. A unique case of transient spontaneous regression complicated with tumor lysis syndrome of T-cell lymphoblastic lymphoma in HIV-infected patient without antiretroviral therapy. J Med Assoc Thai. 2007 Sep;90(9):1930-3. PMID: 17957940.
19	Daly RM, Healy CM, Toner ME, Flint SR. Spontaneous regression of non-Hodgkin's lymphoma in the oral cavity after incisional biopsy. Br J Oral Maxillofac Surg. 2008 Apr;46(3):223-225. doi: 10.1016/j.bjoms.2007.03.010. Epub 2007 May 2. PMID: 17478018.
20	Mulder, D. C., Rosenberg, A. J. W. P., Storm-Bogaard, P. W., & Koole, R. (2010). Spontaneous regression of advanced Merkel-cell-like small cell carcinoma of the parotid gland. British Journal of Oral and Maxillofacial Surgery, 48(3), 199-200.
21	Brachet P, de Leval L, Chantrain G, Loeb I, Saussez S. Régression spontanée d'un lymphome B à grandes cellules du palais [Hard palate B cell lymphoma with spontaneous regression]. Rev Stomatol Chir Maxillofac. 2011 Jun;112(3):180-2. French. doi: 10.1016/j.stomax.2011.01.011. Epub 2011 Apr 8. Erratum in: Rev Stomatol Chir Maxillofac. 2012 Jun;113(3):199. Deleval, L [corrected to de Leval, L]. PMID: 21481900.
22	Corti M, Villafañe MF, Bistmans A, Campitelli A, Narbaitz M, Baré P. Oral cavity and extra-oral plasmablastic lymphomas in AIDS patients: report of five cases and review of the literature. Int J STD AIDS. 2011 Dec;22(12):759-63.
23	Tamás L, Sári E, Répássy G, Szabó P, Bagdi E, Krenács L, Demeter J. Spontaneous remission in localized diffuse large B-cell lymphoma. Pathol Oncol Res. 2011 Sep;17(3):779-84. doi: 10.1007/s12253-011-9379-6. Epub 2011 Jun 10. PMID: 21660524.
24	Fitzpatrick, S. G., Bowers, L. M., Al-Quran, S. Z., Fox, E. G., Cohen, D. M., & Bhattacharyya, I. ALK negative anaplastic large cell lymphoma of the oral cavity showing spontaneous regression.
25	García-Noblejas, A., Velasco, A., Cannata-Ortiz, J., & Arranz, R. (2012). Spontaneous regression of immunodeficiency associated plasmablastic lymphoma related to methotrexate after decrease of dosage. Medicina Clinica, 140(12), 569-570.
26	Choi, N., Cho, J. K., Baek, C. H., Ko, Y. H., & Jeong, H. S. (2014). Spontaneous regression of metastatic cancer cells in the lymph node: a case report. BMC research notes, 7(1), 1-6.
27	de Andrade Sousa A, Lopes Rena R, Souza Silva G, Marcos Arantes Soares J, Porcaro-Salles JM, Nunes L, Alves Mesquita R, Jham BC. Spontaneous remission of a squamous cell carcinoma of the floor of the mouth. J Craniomaxillofac Surg. 2014 Oct;42(7):1536-9.
28	Cuenca-Jimenez, T., Blanco-Guzman, M., & Holwill, S. (2015). Case report: extensive spontaneous regression of metastatic parotid keratinizing squamous cell carcinoma. British Journal of Oral and Maxillofacial Surgery, 53(10), e48-e49.
29	Igawa T, Sato Y, Kawai H, Kondo E, Takeuchi M, Miyata-Takata T, Takata K, Yoshino T. Spontaneous regression of plasmablastic lymphoma in an elderly human immunodeficiency virus (HIV)-negative patient. Diagn Pathol. 2015 Oct 6;10:183. doi: 10.1186/s13000-015-0421-y. PMID: 26445485; PMCID: PMC4596369.
30	Kaibuchi, N., Okamoto, T., Kataoka, T., Kumasaka, A., & Ando, T. (2015). A case of spontaneous regression of lymphoma in the mandibular gingiva after biopsy. Oral and Maxillofacial Surgery Cases, 1, 33-37.
31	Gonzalez-Perez, Luis Miguel & Borrero-Martín, Juan Jose. (2015). An elderly man with a gingival mass that spontaneously regressed. Oral Surgery, Oral Medicine, Oral Pathology, Oral Radiology. 121. 348-352.
32	Wagner VP, Ortiz L, da Silva HP, Meurer L, da Cunha Filho JJ, Martins MA, Martins MD. Impact of highly active antiretroviral therapy in the development and remission of oral plasmablastic lymphoma. Indian J Dent Res. 2016 Sep-Oct;27(5):559-562.
33	Daroit, Natália & Silva, Viviane & Maraschin, Bruna & Visioli, Fernanda & Aleixo, Pedro & Oliveira, Márcia & Rados, Pantelis. (2017). Spontaneous Regression of an Oral Manifestation of Plasmablastic Lymphoma: Literature Review and Commentary about the Phenomena. British Journal of Medicine and Medical Research. 21. 1-7.
34	Kitamura, N., Mizobuchi, T., Ohno, S., Yoshizawa, Y., & Yamamoto, T. (2017). A case of AIDS-related plasmablastic lymphoma of the upper gingiva. Journal of Japanese Society of Oral Oncology, 29(4), 233–239.
35	Rajan, R., & Samant, S. (2008). Spontaneous regression of mucosal melanoma. Otol Head Neck Surg, 139(2 Suppl 1), 128.
36	Yao, M., Yoshioka, N., Hasegawa, K., Shimo, T., & Sasaki, A. (2017). A case of spontaneous regression of plasmablastic lymphoma in the upper gingiva. International Journal of Oral and Maxillofacial Surgery, 46, 315.
37	Miyagawa, F., Ogawa, K., & Asada, H. (2017). A case of CD4+/CD8+ double-positive primary cutaneous anaplastic large cell lymphoma of the lip involving spontaneous regression after biopsy. European Journal of Dermatology, 27(1), 68-69.
38	M F Vargas Gamarra, O Natsuki, M Flores, M Armengot Carceller, Spontaneous partial regression of low-grade mucoepidermoid carcinoma of the maxilla, Oxford Medical Case Reports, Volume 2018, Issue 7, July 2018, omy026
39	Ono, K., Okui, T., Ibaragi, S., Kawai, H., Obata, K., Fujita, M., & Sasaki, A. (2019). A case of intraoral plasmablastic lymphoma spontaneously regressed after biopsy in HIV-negative patient. Journal of Oral and Maxillofacial Surgery, Medicine, and Pathology.
40	Curioni OA, Andrade Filho P, Prates AJ, Rapoport A, Dedivitis RA. Spontaneous regression of adenocarcinoma of submandibular gland. Braz J Otorhinolaryngol. 2021 Jul-Aug;87(4):486-488. doi: 10.1016/j.bjorl.2020.10.014. Epub 2020 Nov 22. PMID: 33303413.
41	de Oliveira, A. A., Lee, I., & Teixeira, G. V. (2020). Spontaneous regression of oral mucosal malignant melanoma. Archives of Head and Neck Surgery, 49, 0-0.
42	Peeters M, Geusens J, Van der Cruyssen F, Michaux L, de Leval L, Tousseyn T, Vandenberghe P, Politis C. Case Report: Spontaneous Remission of an Infraorbital Follicular B-Cell Lymphoma: Case Report and Review of the Literature. Pathol Oncol Res. 2021 Apr 8;27:642433. doi: 10.3389/pore.2021.642433. PMID: 34257608; PMCID: PMC8262163.
43	Aoki Y, Hasegawa S, Miyabe S, Nagao T. Spontaneous regression of malignant lymphoma of the maxillary gingiva following biopsy. Int J Oral Maxillofac Surg. 2021 Sep 21:S0901-5027(21)00326-X. doi: 10.1016/j.ijom.2021.09.006. Epub ahead of print. PMID: 34561111.
44	Lau, K., Lee, C., Tustin, H., & Stafford, F. (2021). Spontaneous regression of advanced stage oropharyngeal squamous cell carcinoma. The Journal of Laryngology & Otology, 1-8. doi:10.1017/S0022215121002899
45	Ueno, S., Yamada, K., Kamitani, M., & Shima, M. (2021). A Case of Oral Malignant Melanoma with Spontaneous Partial Regression. Japanese Journal of Oral Diagnosis / Oral Medicine, 34(3), 208–213.
46	Sousa LG, McGrail DJ, Li K, Marques-Piubelli ML, Gonzalez C, Dai H, Ferri-Borgogno S, Godoy M, Burks J, Lin SY, Bell D, Ferrarotto R. Spontaneous tumor regression following COVID-19 vaccination. J Immunother Cancer. 2022 Mar;10(3):e004371. doi: 10.1136/jitc-2021-004371. PMID: 35241495.
47	Rees, G.J.; Ross, C.M. Abscopal regression following radiotherapy for adenocarcinoma. Br J Radiol 1983, 56, 63-66, doi:10.1259/0007-1285-56-661-63.
48	Ohwada S, ~iyamoto Y, Fujii T, Oyama T, Joshita T, Izuo~. Spontaneous regression of esophageal carci- noma with pulmonary metastases: Case report. Jpn J Clin Onco11990; 20: 193-8.
49	Vergeau B, ~olinie C, Grandpierre G, Vindrios J. Spontaneous partial elimination of a carcinoma of the esophagus. Gastrointest Endosc 1991; 37: 591.
50	HAHM, K. B., SHIM, Y. J., YIM, D. S., KIM, W. H., MOON, Y. M., KANG, J. K., ... & PARK, C. I. (1993). Spontaneous Regression of Primary Malignant Lymphoma of the Esophagus. Korean Journal of Gastrointestinal Endoscopy, 335-339.
51	Saruki, K. et al. A Case of Spontaneous Regression of the Esophageal Cancer during Endoscopic Follow in 2 Years. Prog. Dig. Endosc. 45, 114–116 (1994).
52	Takemura, Osugi, Tokuhara, Kinoshita & Higashino. Case of spontaneous regression of metastatic lesions of leiomyosarcoma of the esophagus. Dis. Esophagus 12, 317–320 (1999).
53	Chang WYM (2000) Complete spontaneous regression of cancer: four case reports, review of literature, and discussion of possible mechanisms involved. Hawaii Med J 59:379–387
54	Kubota, M. et al. Spontaneous regression in small cell esophageal carcinoma. Jpn. J. Thorac. Cardiovasc. Surg. 51, 660–664 (2003).
55	Hornby, S. T., Newman, P. A., Hassan, U. & Robb, W. B. Spontaneous Regression of Malignant Melanoma of the Gastro-Oesophageal Junction. Am. J. Cancer Case Rep. 3, 154–160 (2015).
56	Mitchell R, Kaur A, Munoh Kenne F, Khan A, Zafar W. Spontaneous Regression of Metastatic Lesions of Adenocarcinoma of the Gastro-Esophageal Junction. Cureus. 2021 Oct 14;13(10):e18784. doi: 10.7759/cureus.18784. PMID: 34796071; PMCID: PMC8590531.
57	Khan, H., Casey, P., Hayes, S., Tokala, A., & Sultan, J. (2021). Spontaneous regression of oesophageal squamous cell carcinoma. BMJ Case Reports CP, 14(6), e241344.
58	Takeuchi T, Itoh M et al. A case of early gastric reticulum cell sarcoma : follow-up study with x-ray and gastroendoscope during the early stage. I to Cho (Stomach and Intestine) 1971;6:211-9. (in Japanese)
59	Nakano H, Nakazawa S, Itoh Z et al. A case of reticulum cell sarcoma of the stomach. I to Cho (Stomach and Intestine) 1972 ; 7 : 375-82. (in Japanese)
60	Rosenberg, S. A., Fox, E. & Churchill, W. H. Spontaneous regression of hepatic metastases from gastric carcinoma. Cancer 29, 472–474 (1972).
61	Ohashi Y, Kumakura K, Sugiyama N et al. Early reticulum cell sarcoma of the stomach: report of a case showing remarkable changes within a short time. I to Cho (Stomach and Intestine) 1973;8:195- 203. (in Japanese)
62	Tietjen GW, McAllister FF. Spontaneous regression of gastric reticulum cell sarcoma. N Y State J Med. 1974 Apr;74(4):680-3. PMID: 4596541.
63	Yamazaki S, Nakaizumi H, Shirasaki S. A case of reticulum cell sarcoma of the stomach undergoing changes within a short time. I to Cho (Stomach and Intestine) 1974 ; 9 : 51-5. (in Japanese)
64	Kimura W, Ohtsubo K, Miura H, Kino K. Spontaneous regression of a carcinoma of the residual stomach with latent carcinoma of the pancreas: autopsy report. Jpn J Clin Oncol. 1987 Jun;17(2):187-92. PMID: 3613142.
65	Strauchen JA, Moran C, Goldsmith M, Greenberg M. Spontaneous regression of gastric lymphoma. Cancer. 1987 Oct 15;60(8):1872-5. doi: 10.1002/1097-0142(19871015)60:8<1872::aid-cncr2820600833>3.0.co;2-6. PMID: 3652015.
66	Strauchen JA, Moran C, Goldsmith M, Greenberg M. Spontaneous regression of gastric lymphoma. Cancer. 1987 Oct 15;60(8):1872-5. doi: 10.1002/1097-0142(19871015)60:8<1872::aid-cncr2820600833>3.0.co;2-6. PMID: 3652015.
67	Harvey, R. (1988). Spontaneous resolution of multifocal gastric enterochromaffin-like cell carcinoid tumours. The Lancet, 331(8589), 821.
68	Harvey, R. (1988). Spontaneous resolution of multifocal gastric enterochromaffin-like cell carcinoid tumours. The Lancet, 331(8589), 821.
69	Sawant PD, Nanivadekar SA, Shroff CP, Srinivas A, Dewoolkar VV. Spontaneous regression of large gastric carcinoid. Indian J Gastroenterol. 1989 Oct;8(4):289-90. PMID: 2689331.
70	Shigematsu A, Iida M, Lien GS, Imamura T, Okada M, Fuchigami T, Fujishima M, Itoh H, Iwashita A. Spontaneous regression of primary malignant lymphoma of the stomach in two nontreated Japanese. J Clin Gastroenterol. 1989 Oct;11(5):511-7. doi: 10.1097/00004836-198910000-00006. PMID: 2794430.
71	Shigematsu A, Iida M, Lien GS, Imamura T, Okada M, Fuchigami T, Fujishima M, Itoh H, Iwashita A. Spontaneous regression of primary malignant lymphoma of the stomach in two nontreated Japanese. J Clin Gastroenterol. 1989 Oct;11(5):511-7. doi: 10.1097/00004836-198910000-00006. PMID: 2794430.
72	Rebollo J, Llorente I, Yoldi A. Regresión tumoral espontánea en un paciente con cáncer gástrico metastásico. Communicación de un caso adicional [Spontaneous tumor regression in a patient with metastatic gastric cancer. Communication of an additional case]. Rev Med Univ Navarra. 1990 Jul-Sep;34(3):141-2. Spanish. PMID: 2151656.
73	Yoshimine T, Hamada T, Kubota H et aE. Primary malignant lymphoma of the stomach with spontane- ous regression, report of a case. I to Cho (Stomach and Intestine) 1991;26 :814-8.
74	HAYAKAWA, K., FUKUCHI, S., YOSHIDA, Y., HASHIMOTO, M., HOSHIHARA, Y., MIYAKOSHI, S., ... & UNAKAMI, M. (1992). A Case of Spontaneous Regression of Primary Malignant Lymphoma of the Stomach. Digestive Endoscopy, 4(3), 267-273.
75	Takehara Y, Haruma K, Tsuda T, Sumii K, Kajiyama G. Apparent spontaneous regression of primary malignant lymphoma of the stomach. Endoscopy. 1992 Oct;24(8):735-6. doi: 10.1055/s-2007-1010573. PMID: 1425471.
76	MATSUSAKI, K., KAWANO, T., MIURA, O., TODA, T., MINAMISONO, Y., NAGASAKI, S., & YAO, T. (1996). Primary malignant lymphoma of the stomach with a complete and spontaneous regression in a short period-a case report. The journal of the Japanese Practical Surgeon Society, 57(9), 2198-2202.
77	Ogawa D, Uemura N, Sasaki N, et al. Spontaneous regression of malignant lymphoma of the stomach. Journal of Medicine. 1998 ;29(5-6):381-393. PMID: 10503173.
78	Bariol C, Field A, Vickers CA, et al (2001) Regression of gastric T cell lymphoma with eradication of Helicobacter pylori. Gut 48:269–271
79	Salam, I., Durai, D., Murphy, J. K., & Sundaram, B. (2001). Regression of primary high-grade gastric B-cell lymphoma following Helicobacter pylori eradication. European journal of gastroenterology & hepatology, 13(11), 1375-1378.
80	Pentimone F, Moncini C, Pastine F, Gerini A, Lucchesi Q. Spontaneous regression and recurrence of primary low-grade B-cell gastric lymphoma on the gastric stump 15 and 20 years after gastroresection. Panminerva Med. 2002 Sep;44(3):271-4. PMID: 12094145.
81	Chung, H. W., Son, D. K., Ji, J. S., Chung, D. Y., Chung, J. S., Kim, J. I., ... & Sun, H. S. (2003). A case of spontaneous regression of advanced gastric cancer. Korean Journal of Gastrointestinal Endoscopy, 216-219.
82	Watanabe N, Okazaki K, Yazumi S, Ohana M, Uchida K, Chiba T. Spontaneous regression of Epstein-Barr virus-associated T-cell lymphoma of the stomach. Gastrointest Endosc. 2003 Mar;57(3):414-7. doi: 10.1067/mge.2003.132. PMID: 12612533.
83	Watari, J., Saitoh, Y., Fujiya, M. et al. Spontaneous remission of primary diffuse large B-cell gastric lymphoma. J Gastroenterol 40, 414–420 (2005).
84	Watari, J., Saitoh, Y., Fujiya, M. et al. Spontaneous remission of primary diffuse large B-cell gastric lymphoma. J Gastroenterol 40, 414–420 (2005).
85	Ohno Y, Kosaka T, Muraoka I, et al. Remission of primary low-grade gastric lymphomas of the mucosa-associated lymphoid tissue type in immunocompromised pediatric patients. World J Gastroenterol 2006;12:2625-2628.
86	Lee HS, Cheung DY, Kim JI, Cho SH, Park SH, Han JY, Kim JK. A case of spontaneous regression of advanced gastric cancer. J Korean Med Sci. 2010 Oct;25(10):1518-21. doi: 10.3346/jkms.2010.25.10.1518. Epub 2010 Sep 20. PMID: 20890436; PMCID: PMC2946665.
87	Ip, Y.-T., Pong, W.-M., Kao, S.-S. & Chan, J. K. C. Spontaneous Complete Regression of Gastric Large-Cell Neuroendocrine Carcinoma: Mediated by Cytomegalovirus-Induced Cross-Autoimmunity? Int. J. Surg. Pathol. 19, 355–358 (2011).
88	A Patient with Distinct Evidences of Spontaneous Regression and Recurrence of Gastric Cancer for 13 Years. (2012). Ann Geriatr Med Res, 16(3), 149–152. doi:10.4235/jkgs.2012.16.3.149
89	Rojas-Hernandez, C. M., Coleman, J. F., Czuchlewski, D. R. & Fekrazad, M. H. Spontaneous regression of high grade primary gastric Lymphoma in an untreated viral hepatitis infection. Leuk. Lymphoma 55, 2643–2645 (2014).
90	Shibata, M., Matsubayashi, H., Todaka, A., Kurai, H., Tsutsumi, N., Sasaki, K., & Ono, H. (2016). The Disappearance of Lymph Node Metastasis from Neuroendocrine Carcinoma after Endoscopic Ultrasound-guided Fine Needle Aspiration. Internal Medicine, 55(19), 2805–2809.
91	Sugiyama, T., Arita, K., Shinno, E., & Nakajima, T. (2018). Spontaneous remission of diffuse large B cell lymphoma in the stomach and the continuation of remission for 10 years. Case reports in gastroenterology, 12(3), 699-703.
92	Bonilla, C.E.; Esguerra, J.; Mendoza Díaz, S.; Álvarez, A.; Morales, R.L. Abscopal Effect after Palliative Radiotherapy in a Patient with a Gastric Adenocarcinoma Disseminated to Retroperitoneal Space: Case Report from a Latin American Reference Center and Review of the Literature. Cureus 2019, 11, e6235.
93	Hatsuse M, Ide D, Matsui S, Fuchida SI, Murakami S, Shimazaki C. [Spontaneous regression of primary gastric EBV-positive diffuse large B-cell lymphoma]. Rinsho Ketsueki. 2019;60(11):1573-1576. Japanese.
94	Okamoto T, Yoshimoto T, Ohike N, Fujikawa A, Kanie T, Fukuda K. Spontaneous regression of gastric gastrinoma after resection of metastases to the lesser omentum: A case report and review of literature. World J Gastroenterol. 2021 Jan 7;27(1):129-142. doi: 10.3748/wjg.v27.i1.129. PMID: 33505155; PMCID: PMC7789063.
95	Zafar M, Paracha AW, Ashraf M, Muhammad T, Whitehead M, Toqeer M. Delayed Spontaneous Regression of Metastatic Gastric Cancer: A Case Report of a Rare Finding. Cureus. 2021 Dec 7;13(12):e20224. doi: 10.7759/cureus.20224. PMID: 34900506; PMCID: PMC8649674.
96	Schwartz, E., Maayan, C., Mouallem, M., Engelberg, S., & Friedman, E. (1991). Malignant peritoneal mesothelioma: Long‐term spontaneous clinical remission. Medical and pediatric oncology, 19(4), 325-328.
97	BaniHani MN, Al Manasra AR. Spontaneous regression in alveolar soft part sarcoma: case report and literature review. World J Surg Oncol. 2009 Jun 10;7:53. doi: 10.1186/1477-7819-7-53. PMID: 19515237; PMCID: PMC2703639.
98	Jagodic, M. (2010, July). 3. P. 35 “SPONTANEOUS” REGRESSION OF ADVANCED RETROPERITONEAL LEIOMYOSARCOMA AFTER THE END OF SALVAGE CHEMOTHERAPY. In Orthopaedic Proceedings (Vol. 92, No. SUPP_III, pp. 447-447). The British Editorial Society of Bone & Joint Surgery.
99	Gottfried EB, Steller R, Paronetto F, Lieber CS. Spontaneous regression of hepatocellular carcinoma. Gastroenterology 1982; 82:770– 4.
100	Lam KC, Ho JC, Yeung RT. Spontaneous regression of hepatocellular carcinoma: a case study. Cancer. 1982 Jul 15;50(2):332-6. doi: 10.1002/1097-0142(19820715)50:2<332::aid-cncr2820500228>3.0.co;2-o. PMID: 6282440.
101	McCaughan GW, Bilous MJ, Gallagher ND (1985) Long-term survival with tumor regression in androgen-induced liver tumors. Cancer 56:2622–2626
102	McCaughan GW, Bilous MJ, Gallagher ND (1985) Long-term survival with tumor regression in androgen-induced liver tumors. Cancer 56:2622–2626
103	Sato Y, Fujiwara K, Nakagawa S, Kanishima S, Ohta Y, Oka Y, Hayashi S, Oka H. A case of spontaneous regression of hepatocellular carcinoma with bone metastasis. Cancer. 1985 Aug 1;56(3):667-71. doi: 10.1002/1097-0142(19850801)56:3<667::aid-cncr2820560339>3.0.co;2-s. PMID: 2408740.
104	Takayasu K, Muramatsu Y, Shima Y, Moriyama N, Yamada T, Yoshida T, Makuuchi M, Kishi K. Necrosis of hepatocellular carcinoma as a result of subintimal injury incurred by hepatic angiography: report of two cases. Am J Gastroenterol. 1986 Oct;81(10):979-83. PMID: 3020974.
105	Takayasu K, Muramatsu Y, Shima Y, Moriyama N, Yamada T, Yoshida T, Makuuchi M, Kishi K. Necrosis of hepatocellular carcinoma as a result of subintimal injury incurred by hepatic angiography: report of two cases. Am J Gastroenterol. 1986 Oct;81(10):979-83. PMID: 3020974.
106	Andreola S, Audisio RA, Mazzaferro V, Doci R, Milella M: Spontaneous massive necrosis of a hepatocellular carcinoma. Tumori 1987;73:203–207.
107	Royuela, S.F., Prolonged course of hepatocellular carcinoma A spontaneous regression. Gastroenterlogia Y. Hepatologia, 1989. 12(10).
108	Suzuki, M., et al., Spontaneous Regression of Hepatocellular Carcinoma--a case report, Suzuki M, Okazaki, Hepatogastroenterology, 1989. pdf. New York, 1989. 36: p. 160-163.
109	Ayres, R.C., et al., Spontaneous regression of hepatocellular carcinoma. Gut, 1990. 31(6): p. 722-4.
110	Gaffet, M.J. and J.P. Joyce, . Cancer, 1990.
111	Tocci, G., et al., Spontaneous remission of hepatocellular carcinoma after massive gastrointestinal haemorrhage Coffee consumption as trigger for insulin-dependent diabetes mellitus in childhood. 1988. 300.
112	Mochizuki, T. and Y. Takehara, Regression of hepatocellular carcinoma. AJR, 1991.
113	Masakazu Yamamoto, Ken Takasaki, Takaho Watayō, et al. Spontaneous Necrosis Due to a Hepatocellular Carcinoma. Cancer clinical 1991; 37: 491-496
114	Masakazu Yamamoto, Ken Takasaki, Takaho Watayō, et al. Spontaneous Necrosis Due to a Hepatocellular Carcinoma. Cancer clinical 1991; 37: 491-496
115	Chien, R.-N., T.J. Chen, and Y.-F. Liaw, Spontaneous Regression of Hepatocellular Carcinoma,Chien RN, Chen TJ, Liaw YF, American Journal Gastroenterology, 1992.pdf. 1992, The American Journal of Gastroenterology.
116	Imaoka, S. and Y. Sasaki, Necrosis of hepatocellular Carcinoma Caused by Spontaneously Arising Arterial Thrombus 1994. Hepato-Gastroenterology, 1994. 41: p. 359-362.
117	McDermott, W.V. and U. Khettry, Clear cell carcinoma of the liver with spontaneous regression of metastases. J Surg Oncol, 1994. 57(3): p. 206-209.
118	Grossmann M, Hoermann R, Weiss M, Jauch KW, Oertel H, Staebler A, Mann K, Engelhardt D. Spon- taneous regression of hepatocellular carcinoma. Am J Gastroenterol 1995; 90: 1500-1503
119	Herrera A, Erdozain JC, Molina E, Conde P, Palomo V. Spontaneous regression of hepatocellular carcinoma. Am J Gastroenterol 1996; 91:1288 – 1289.
120	Ozeki Y, Matsubara N, Tateyama K, Kokubo M, Shimoji H, Katayama M. Spontaneous complete necrosis of hepatocellular carcinoma. Am J Gastroenterol. 1996;91:391–2.
121	Markovic S, Ferlan-Marolt V, Hlebanja Z. Spontaneous regression of hepatocellular carcinoma. Am J Gastroenterol. 1996;91:392–3.
122	Yoshimitsu, K., Honda, H., Kaneko, K., Fukuya, T., Irie, H., Aibe, H., ... & Masuda, K. (1996). Temporary spontaneous regression of intrahepatic cholangiocarcinoma. Computerized medical imaging and graphics, 20(2), 115-118.
123	Iwasaki, M., et al., Spontaneous regression of hepatocellular carcinoma: a case report. Japanese journal of clinical oncology, 1997. 27(4): p. 278-281.
124	Van Halteren, H.K., J.M.J.I. Salemans, and H. Peters. Journal of Hepatology, 1997. 27: p. 211-215.
125	Kaczynski, J., et al., Spontaneous regression of hepatocellular carcinoma Case report. 1998: p. 147-150.
126	Ohba, K., et al., Abscopal regression of hepatocellular carcinoma after radiotherapy for bone metastasis. Gut, 1998. 43(4): p. 575-577.
127	Magalotli, D., C. Gueli, and M. Zoli, Transient spontaneous regression of hepatocellular carcinoma. 1998. p. 2369-2371.
128	Magalotli, D., C. Gueli, and M. Zoli, Transient spontaneous regression of hepatocellular carcinoma. 1998. p. 2369-2371.
129	Gómez Sanz R, Moreno Gonzalez E, Colina Ruiz- Delgado F, Garcia-Muñoz H, Ochando Cerdan F, Gonzalez-Pinto I. Spontaneous regression of a re- current hepatocellular carcinoma. Dig Dis Sci 1998; 43: 323-328
130	Stoelben E, Koch M, Hanke S, Lossnitzer A, Gaert- ner HJ, Schentke KU, Bunk A, Saeger HD. Spontaneous regression of hepatocellular carcinoma confirmed by surgical specimen: report of two cases and review of the literature. Langenbecks Arch Surg 1998; 383: 447-452
131	Stoelben E, Koch M, Hanke S, Lossnitzer A, Gaert- ner HJ, Schentke KU, Bunk A, Saeger HD. Spontaneous regression of hepatocellular carcinoma confirmed by surgical specimen: report of two cases and review of the literature. Langenbecks Arch Surg 1998; 383: 447-452
132	Takeuchi, K., Nishino, H., Ohira, M., & Sowa, M. (1998). A case of hepatocellular carcinoma with massive spontaneous necrosis presented unusual image. Nihon Rinsho Geka Gakkai Zasshi (Journal of Japan Surgical Association), 59(6), 1614–1618.
133	Itoh, T., Kanamaru, T., Morita, Y., Yamamoto, M., Kuroda, Y., Hayashi, Y., & Itoh, H. (1999). A Case Report of Spontaneous Complete Necrosis of Hepatocellular Carcinoma. The Japanese Journal of Gastroenterological Surgery, 32(1), 32–35.
134	Toyoda H, Sugimura S, Fukuda K, Mabuchi T. Hepa- tocellular carcinoma with spontaneous regression of multiple lung metastases. Pathol Int 1999; 49: 893-897
135	Izuishi K, Ryu M, Hasebe T, Kinoshita T, Konishi M, Inoue K. Spontaneous total necrosis of hepatocellular carcinoma. Hepatogastroenterology. 2000;47:1122–4.
136	Jang TJ, Lee JI, Kim DH, Kim JR, Lee HK. Spontane- ous regression of hepatocellular carcinoma--a case report. Korean J Intern Med 2000; 15: 147-150
137	Lee HS, Lee JS, Woo GW, Yoon JH, Kim CY. Recur- rent hepatocellular carcinoma after spontaneous regression. J Gastroenterol 2000; 35: 552-556
138	Lee HS, Lee JS, Woo GW, Yoon JH, Kim CY. Recur- rent hepatocellular carcinoma after spontaneous regression. J Gastroenterol 2000; 35: 552-556
139	Takeda Y, Togashi H, Shinzawa H, Miyano S, Ishii R, Karasawa T, Takeda Y, Saito T, Saito K, Haga H, Matsuo T, Aoki M, Mitsuhashi H, Watanabe H, Takahashi T. Spontaneous regression of hepatocellular carcinoma and review of literature. J Gastroen- terol Hepatol 2000; 15: 1079-1086
140	Terasaki T, Hanazaki K, Shiohara E, Matsunaga Y, Koide N, Amano J. Complete disappearance of recurrent hepatocellular carcinoma with peritoneal dissemination and splenic metastasis: a unique clinical course after surgery. J Gastroenterol Hepatol 2000; 15: 327-330
141	Uenishi T, Hirohashi K, Tanaka H, Ikebe T, Kinoshi- ta H. Spontaneous regression of a large hepatocellular carcinoma with portal vein tumor thrombi: report of a case. Surg Today 2000; 30: 82-85
142	Ikeda M, Okada S, Ueno H, et al. Spontaneous regression of hepatocellular carcinoma with multiple lung metastases: a case report. Jpn J Clin Oncol 2001; 31 (9): 454―458
143	Jung, D. Y., Kim, Y. B., Lee, Y. H., Lee, Y. K., Kim, J. R., Bae, Y. O., & Cho, I. S. (2001). A Case of Spontaneous Regression of Metastatic Hepatocellular Carcinoma. The Korean Journal of Gastroenterology, 38(6), 436-439.
144	Kawai, M., Hara, H., Dohi, T., Morita, S., Tanigawa, N., Takeshita, A., … Shibayama, Y. (2001). Spontaneous complete coagulative necrosis of hepatocellular carcinoma. Kanzo, 42(9), 471–476.
145	Matsuo R, Ogata H, Tsuji H, Kitazono T, Shimada M, Taguchi K, et al. Spontaneous regression of hepatocellular carcinoma. Hepatogastroenterology. 2001 Nov–Dec;48(42):1740–2.
146	Nakai T, Shimomura T, Hirokawa F. Spontaneous 33 regression of recurrent hepatocellular carcinoma after TAE: possible mechanisms of immune mediation. Int J Clin Oncol 2001; 6: 149-152
147	Sakurai Y, Shoji M, Matsubara T, Suganuma M, Hasegawa S, Imazu H, Ochiai M, Funabiki T, Urano M, Mizoguchi Y, et al. Spontaneous necrosis of gallbladder carcinoma in patient with pancreaticobiliary maljunction. J Hepatobiliary Pancreat Surg. 2001;8:95–100.
148	Garrido Serrano A, Guerrero Igea FJ, Lepe Jiménez JA, Palomo Gil S. [Spontaneous regression of hepatocellular carcinoma in a cirrhotic patient]. Gastro- enterol Hepatol 2001; 24: 503-505
149	Abiru S, Kato Y, Hamasaki K, Nakao K, Nakata K, Eguchi K. Spontaneous regression of hepatocellular carcinoma associated with elevated levels of inter- leukin 18. Am J Gastroenterol 2002; 97: 774-775
150	Abiru S, Kato Y, Hamasaki K, Nakao K, Nakata K, Eguchi K. Spontaneous regression of hepatocellular carcinoma associated with elevated levels of inter- leukin 18. Am J Gastroenterol 2002; 97: 774-775
151	Abiru S, Kato Y, Hamasaki K, Nakao K, Nakata K, Eguchi K. Spontaneous regression of hepatocellular carcinoma associated with elevated levels of inter- leukin 18. Am J Gastroenterol 2002; 97: 774-775
152	Lee SC, Chung HW, Chung JB, Park YN, Ahn SH, Park SW, Chun CY, Moon YM, Kang JK, Park IS. Total necrosis of hepatocellular carcinoma due to spontaneous occlusion of feeding artery. Yonsei Med J 2002; 43: 123-127
153	Misawa K, Hata Y, Manabe K, Matsuoka S, Saito M, Takada J, Sano F. Spontaneous regression of hepatocellular carcinoma. J Gastroenterol 1999; 34: 410-414
154	Morimoto Y, Tanaka Y, Itoh T, Yamamoto S, Mizuno H, Fushimi H. Spontaneous necrosis of hepatocellular carcinoma. Dig Surg. 2002;19:413–8.
155	Zimmermann, A., Kappeler, A., Friess, H., & Büchler, M. W. (2002). Hepatocellular carcinoma with an unusual medullary-like histology and signs of regression (“medullary-like hepatocellular carcinoma”). Digestive and Liver Disease, 34(10), 748-753.
156	Iiai T, Sato Y, Nabatame N, Yamamoto S, Makino S, Hatakeyama K. Spontaneous complete regression of hepatocellular carcinoma with portal vein tumor thrombus. Hepatogastroenterology. 2003 Sep–Oct;50(53):1628–30.
157	Jozuka H, Jozuka E, Suzuki M, Takeuchi S, Takatsu Y . Psycho-neuro-immunological treatment of hepatocellular carcinoma with major depression--a single case report. Curr Med Res Opin 2003; 19: 59-63
158	Li AJ, Wu MC, Cong WM, Shen F, Yi B. Spontaneous complete necrosis of hepatocellular carcinoma. Hepatobiliary Pancreat Dis Int. 2003 Feb;2(1):152–4.
159	Ohta H, Sakamoto Y, Ojima H, Yamada Y, Hibi T, Takahashi Y, et al. Spontaneous regression of hepatocellular carcinoma with complete necrosis. Abdom Imaging. 2005 Nov–Dec;30(6):734–7.
160	Blondon H, Fritsch L, Cherqui D. Two cases of spontaneous regression of multicentric hepatocellular carcinoma after intraperitoneal rupture: possible role of immune mechanisms. Eur J Gastroenterol Hepatol 2004; 16 (12): 1355―1359
161	Blondon H, Fritsch L, Cherqui D. Two cases of spontaneous regression of multicentric hepatocellular carcinoma after intraperitoneal rupture: possible role of immune mechanisms. Eur J Gastroenterol Hepatol 2004; 16 (12): 1355―1360
162	Cheng HM, Tsai MC. Regression of hepatocellular carcinoma spontaneous or herbal medicine related? Am J Chin Med 2004; 32 (4): 579―585
163	Erturk, S., Yuceyar, S., Yigitbasi, R., Onur, E., Saner, H., & Uygun, N. (2004). Spontaneous regression of hepatocellular carcinoma. A case report. CHIRURGIA-TORINO-, 17(3), 95-98.
164	Feo CF, Marrosu A, Scanu AM, et al. Spontaneous regression of hepatocellular carcinoma: report of a case. Eur J Gastroenterol Hepatol 2004; 16 (9): 933― 936
165	Kato H, Nakamura M, Muramatsu M, Orito R, Ueda R, Mizokami M. Spontaneous regression of hepatocellular carcinoma: two case reports and a literature review. Hepatol Res 2004; 29:180-190
166	Kato H, Nakamura M, Muramatsu M, Orito R, Ueda R, Mizokami M. Spontaneous regression of hepatocellular carcinoma: two case reports and a literature review. Hepatol Res 2004; 29:180-190
167	Lin TJ, Liao LY, Lin CL, et al. Spontaneous regression of hepatocellular carcinoma: a case report and literature review. Hepatogastroenterology 2004; 51 (56): 579―582
168	Nakajima T, Moriguchi M, Watanabe T, et al. Recurrence of hepatocellular carcinoma with rapid growth after spontaneous regression. World J Gastroenterol 2004 Nov 15; 10 (22): 3385―3387
169	Jeon SW, Lee MK, Lee YD, et al. Clear cell hepatocellular carcinoma with spontaneous regression of primary and metastatic lesions. Korean J Intern Med 2005; 20 (3): 268―273
170	Moon TG, Joon Hyeok Lee, Moon Seok Choi, Kwang Cheol Koh, Jae J. Kim, Seung Woon Paik, Byung Cheol Yoo, Jong Chul Rhee. A Case of Complete Remission after Multiple Sessions of Local Treatment in Metastatic Hepatocellular Carcinoma. (2006). J Liver Cancer, 6(1), 70–76.
171	Nam SW, Han JY, Kim JI, et al. Spontaneous regression of a large hepatocellular carcinoma with skull metastasis. J Gastroenterol Hepatol 2005 ; 20 ( 3 ) : 488―492
172	Nouso K, Uematsu S, Shiraga K, et al. Regression of hepatocellular carcinoma during vitamin K ad-ministration. World J Gastroenterol 2005; 11 ( 42 ) : 6722―6724
173	Ohtani H, Yamazaki O, Matsuyama M, Horii K, Shimizu S, Oka H, et al. Spontaneous regression of hepatocellular carcinoma. Surg Today. 2005;35(12):1081–6.
174	Randolph AC, Tharalson EM, Gilani N. Spontaneous regression of hepatocellular carcinoma is possible and might have implications for future therapies. Eur J Gastroenterol Hepatol 2008; 20 (8): 804― 809
175	Rizell, M., et al., Impressive regression of primary liver cancer after treatment with sirolimus. Acta Oncologica, 2005. 44(5): p. 496-496.
176	Yano Y, Yamashita F, Kuwaki K, et al. Partial spontaneous regression of hepatocellular carcinoma: a case with high concentrations of serum lens culinaris agglutinin-reactive alpha-fetoprotein. Ku- rume Med J 2005; 52 (3): 97―103
177	Otrock, Z. K., Al-Kutoubi, A., Kattar, M. M., Zaatari, G., & Soweid, A. (2006). Spontaneous complete regression of hepatic epithelioid haemangioendothelioma. The lancet oncology, 7(5), 439-441.
178	Kojima H, Tanigawa N, Kariya S, Komemushi A, Shomura Y, Sawada S, Arai E, Yokota Y. A case of spontaneous regression of hepatocellular carcinoma with multiple lung metastases. Radiat Med. 2006 Feb;24(2):139-42. doi: 10.1007/BF02493281. PMID: 16715676.
179	Kondo S, Okusaka T, Ueno H, et al. Spontaneous regression of hepatocellular carcinoma. Int J Clin Oncol 2006; 11 (5): 407―411
180	Kondo S, Okusaka T, Ueno H, et al. Spontaneous regression of hepatocellular carcinoma. Int J Clin Oncol 2006; 11 (5): 407―411
181	Kondo S, Okusaka T, Ueno H, et al. Spontaneous regression of hepatocellular carcinoma. Int J Clin Oncol 2006; 11 (5): 407―411
182	Kondo S, Okusaka T, Ueno H, et al. Spontaneous regression of hepatocellular carcinoma. Int J Clin Oncol 2006; 11 (5): 407―411
183	Shibuya, K., Bando, T., Onishi, Y., Nagata, T., Yamagishi, F., & Tsukada, K. (2006). A case of spontaneous complete necrosis of hepatocellular carcinoma. Nihon Rinsho Geka Gakkai Zasshi (Journal of Japan Surgical Association), 67(9), 2152–2156.
184	Heianna J, Miyauchi T, Suzuki T, Ishida H, Hashi- moto M, Watarai J. Spontaneous regression of multiple lung metastases following regression of hepatocellular carcinoma after transcatheter arterial embolization. A case report. Hepatogastroenterology 2007; 54: 1560-1562
185	Matsunaga, K., Uesaka, K., Maeda, A., Kanemoto, H., & Furukawa, H. Spontaneous Regression of Sarcomatous Hepatocellular Carcinoma-Report of a Case.
186	Meza-Junco J, Monta!o-Loza AJ, Martinez-Benítez B, et al. Spontaneous partial regression of hepatocellular carcinoma in a cirrhotic patient. Ann Hepatol 2007; 6 (1): 66―69
187	Peddu P, Huang D, Kane PA, et al. Vanishing liver tumours. Clin Radiol 2008; 63 (3): 329―339
188	Vardhana HG, Panda M. Spontaneous regression of hepatocellular carcinoma: potential promise for the future. South Med J 2007; 100 (2): 223―224
189	Arakawa Y, Mori H, Ikegami T, Hanaoka J, Kanamoto M, Kanemura H, et al. Hepatocellular carcinoma with spontaneous regression: report of the rare case. Hepatogastroenterology. 2008 Sep–Oct;55(86–87):1770–2.
190	Hori T, Wagata T, Takemoto K, Shigeta T, Takuwa H, Hata K, Uemoto S, Yokoo N. Spontaneous necrosis of solid gallbladder adenocarcinoma accompanied with pancreaticobiliary maljunction. World J Gastroenterol. 2008 Oct 14;14(38):5933-7.
191	Sibartie V, Moriarty J, Crowe J. Spontaneous regression of hepatocellular carcinoma. Am J Gastroenterol. 2008 Apr;103(4):1050-1. doi: 10.1111/j.1572-0241.2007.01772_14.x. PMID: 18397439.
192	Del Poggio P, Mattiello M, Gilardoni L, et al. The mysterious case of spontaneous disappearance of hepatocellular carcinoma. Dig Liver Dis 2009; 41 (7): e21―25
193	Hsu CY, Sun PL, Chang HC, Perng DS, Chen YS. Spontaneous regression of advanced hepatocellular carcinoma: a case report. Cases J 2009; 2: 6251
194	Kanzaki, A., Hibino, S., Nishi, T., Kawagoe, T., Ito, A., & Sakakibara, S. (2009). A case of spontaneous complete necrosis of hepatocellular carcinoma. Kanzo, 50(5), 244–249.
195	Nishijima N, Marusawa H, Kita R, et al. Education and Imaging. Hepatobiliary and pancreatic: spontaneous regression of hepatocellular cancer demonstrated by contrast-enhanced ultrasonography. J Gastroenterol Hepatol 2009; 24 (6): 1153
196	Oquiñena S, Iñarrairaegui M, Vila JJ, et al. Spontaneous regression of hepatocellular carcinoma: three case reports and a categorized review of the literature. Dig Dis Sci 2009; 54 (5): 1147―1153
197	Oquiñena S, Iñarrairaegui M, Vila JJ, et al. Spontaneous regression of hepatocellular carcinoma: three case reports and a categorized review of the literature. Dig Dis Sci 2009; 54 (5): 1147―1153
198	Oquiñena S, Iñarrairaegui M, Vila JJ, et al. Spontaneous regression of hepatocellular carcinoma: three case reports and a categorized review of the literature. Dig Dis Sci 2009; 54 (5): 1147―1153
199	Park HS, Jang KY, Kim YK, et al. Hepatocellular carcinoma with massive lymphoid infiltration: a regressing phenomenon? Pathol Res Pract 2009; 205 (9): 648―652
200	Harada, T., Sakaguchi, T., Inaba, K., Nakamura, T., Kurachi, K., Fukazawa, A., … Konno, H. (2010). Spontaneous regression of hepatocellular carcinomas. Nippon Shokakibyo Gakkai Zasshi, 107(3), 432–441.
201	Hong JH, Seo DD, Jeon TJ, Oh TH, Shin WC, Choi WC, Cho HS. [A case of spontaneous regression of hepatocellular carcinoma with multiple lung metastases]. Korean J Gastroenterol. 2010 Feb;55(2):133-8. Korean. doi: 10.4166/kjg.2010.55.2.133. PMID: 20168060.
202	Kai, K., Miyoshi, A., Ario, K., Kitahara, K., Mizuta, T., Kawazoe, S., … Miyazaki, K. (2010). The two cases of massive spontaneous necrosis of hepatocellular carcinoma after angiography. Kanzo, 51(6), 312–318.
203	Kai, K., Miyoshi, A., Ario, K., Kitahara, K., Mizuta, T., Kawazoe, S., … Miyazaki, K. (2010). The two cases of massive spontaneous necrosis of hepatocellular carcinoma after angiography. Kanzo, 51(6), 312–318.
204	佐藤政弘. プロスタグランジン合成阻害剤ケトプロフェンにより一過性の腫瘍退縮現象を生じたと考えられる肝細胞癌の1例. 秋田県医師会雑誌 2010; 60: 96
205	Storey RE, Huerta AL, Khan A, Laber DA. Spontaneous complete regression of hepatocellular carcinoma. Med Oncol. 2011 Dec;28(4):948–50.
206	Alqtub A, Peck D, Marotta P. Spontaneous regression of large hepatocellular carcinoma: case report. Ger Med Sci 2011; 9: Doc07
207	Arora, N., & Madhusudhana, S. (2011). Spontaneous regression of hepatocellular cancer: case report and review of literature. Gastrointestinal Cancer Research: GCR, 4(4), 141.
208	Fukushima, H., Shibayama, T., Hishiki, S., Yamamoto, C., & Okada, Y. (2011). A case of hepatocellular carcinoma (HCC) with multiple lung metastases, with disappearance of both the primary HCC and lung metastases after transcatheter arterial chemoembolization (TACE) for the primary HCC and no recurrence for more than 7 years after the TACE.
209	Maejima R, Nakayama H, Horii T, Tosa M, Dairaku N, Shiina M, Dairaku N, Oriuchi T, Ikeda T, Ueno T, Kusano M, Ikeya S, Hiwatashi N. [A case of spontaneous complete necrosis of hepatocellular carcinoma sized 10cm in diameter demonstrated by the resected specimen]. Nihon Shokakibyo Gakkai Zasshi. 2011 Nov;108(11):1902-9.
210	Okano A, Takakuwa H, Nakamura T, et al. Spontaneous regression of diffuse intrahepatic recurrence with portal vein tumor thrombus after resection of hepatocellular carcinoma. Clin J Gastroenterol 2011; 4: 43―48
211	Okuma, K., Yamashita, H., Niibe, Y., Hayakawa, K., & Nakagawa, K. (2011). Abscopal effect of radiation on lung metastases of hepatocellular carcinoma: a case report. Journal of medical case reports, 5(1), 1-4.
212	Bastawrous, S., M.J. Kogut, and P. Bhargava, Spontaneous regression of hepatocellular carcinoma in a cirrhotic patient: Possible vascular hypothesis. Singapore Medical Journal, 2012. 53(10): p. 2-5.
213	Harimoto, N., et al., Spontaneous regression of multiple pulmonary recurrences of hepatocellular carcinoma after hepatectomy: Report of a case. Surgery Today, 2012. 42(5): p. 475-478.
214	Komatsu H, Imamura S, Shimizu T, et al. Clinical Journal of Gastroenterology 2012; 5: 35―41
215	Nakayama S. Spontaneous regression of hepatocellular carcinoma. Indian J Gastroeterol 2012 ; 31 :267―270
216	Takeura C, Tokoro T, Tanahashi Y, Yoshida J, Kagawa T, Kato Y, et al. Two cases of spontaneous regression of hepatocellular carcinoma with extrahepatic metastasis. 2012. Poster session at the 2012 American HepatoPancreatoBiliary Association Annual Meeting, Miami, FL.
217	Takeura C, Tokoro T, Tanahashi Y, Yoshida J, Kagawa T, Kato Y, et al. Two cases of spontaneous regression of hepatocellular carcinoma with extrahepatic metastasis. 2012. Poster session at the 2012 American HepatoPancreatoBiliary Association Annual Meeting, Miami, FL.
218	Yamamoto, S., Tokuhara, T., Nishikawa, M., Nishizawa, S., Nishioka, T., Nozawa, A., … Kubo, S. (2012). Spontaneous regression of hepatocellular carcinoma after improving diabetes mellitus: possibly responsible for immune system. Kanzo, 53(3), 164–174.
219	Yokoyama T, Yoshida H, Hirakata A, Makino H, Maruyama H, Suzuki S, et al. Spontaneous complete necrosis of advanced hepatocellular carcinoma. J Nippon Med Sch. 2012;79(3):213–7.
220	Katayama, Y., Morinaga, S., Numata, M., Godai, T., Masuda, M., & Akaike, M. (2013). A Case of Resection for Complete Spontaneous Necrosis of Hepatocellular Carcinoma. Nihon Rinsho Geka Gakkai Zasshi (Journal of Japan Surgical Association), 74(10), 2863–2868.
221	Okano A, Ohana M, Kusumi F, et al. Spontaneous regression of hepatocellular carcinoma due to dis- ruption of the feeding artery. Case Rep Oncology 2013; 6: 180―185
222	Sasaki, T., Fukumori, D., Yamamoto, K., Yamamoto, F., Igimi, H., & Yamashita, Y. (2013). Management considerations for purported spontaneous regression of hepatocellular carcinoma: a case report. Case Reports in Gastroenterology, 7(1), 147-152.
223	Tomishige, H., Morise, Z., Mizoguchi, Y., Kawabe, N., Nagata, H., Ohshima, H., ... & Isetani, M. (2013). A case of solitary necrotic nodule treated with laparoscopic hepatectomy: spontaneous regression of hepatocellular carcinoma?. Case Reports in Hepatology, 2013.
224	植田 茂, 調 憲, 山下洋市, 他. 1度自然消退するも4年後に再燃した肝細胞癌の1切除例. 臨床と研究 2013; 90: 131-134
225	Bhardwaj, N., Li, M., Price, T., & Maddern, G. J. (2014). Spontaneous regression of a biopsy confirmed hepatocellular carcinoma. Case Reports, 2014, bcr2014204897.
226	F. Chiesara, A. Spagnolo, M. Koch, and A. Moretti, “A case of hepatocellular carcinoma: spontaneous regression?,” Digestive and Liver Disease, vol. 46, no. 7, pp. 659-660, 2014.
227	Masafumi Inoue, Takuya Kimura, Yasuo Matsuda, et al. A case of spontaneous necrosis of hepatocellular carcinoma: possible induction by medication. Rinsho Geka 2014; 69: 377-381
228	Lim, D. H., Park, K. W., & Lee, S. I. (2014). Spontaneous complete regression of multiple metastases of hepatocellular carcinoma: A case report. Oncology letters, 7(4), 1225-1228.
229	Miyake, M., Sugita, M., Mogaki, M., Fukushima, T., Masui, H., Nagahori, K., & Tsuura, Y. (2014). A Case of Spontaneous Complete Necrosis of Hepatocellular Carcinoma. Nihon Rinsho Geka Gakkai Zasshi (Journal of Japan Surgical Association), 75(7), 1972–1978.
230	Saito R, Amano H, Abe T, Fujikuni N, Nakahara M, Yonehara S, Teramen K, Noriyuki T. Complete spontaneous necrosis of hepatocellular carcinoma confirmed on resection: A case report. Int J Surg Case Rep. 2016;22:70-4.
231	Tomino T, Yamashita Y, Iguchi T, et al. Spontane- ous massive necrosis of hepatocellular carcinoma with narrowing and occlusion of the arteries and portal veins. Case Rep Gastroenterol 2014; 8: 148― 155
232	Tsai SC, Kao JL, Shiao CC. Spontaneous regression of a hepatoma with ring calcification. Acta Clin Belg. 2014;69:130–131.
233	Zhao, X.Y., Rakhda, M.I., Habib, S., Bihi, A., Muhammad, A., Wang, T.L., & Jia, J. (2014). Hepatic epithelioid hemangioendothelioma: A comparison of Western and Chinese methods with respect to diagnosis, treatment and outcome. Oncology Letters, 7, 977-983.
234	Parks AL, McWhirter RM, Evason K, Kelley RK. Cases of spontaneous tumor regression in hepatobiliary cancers: implications for immunotherapy? J Gastrointest Cancer. 2015;46:161–165.
235	Parks AL, McWhirter RM, Evason K, Kelley RK. Cases of spontaneous tumor regression in hepatobiliary cancers: implications for immunotherapy? J Gastrointest Cancer. 2015;46:161–165.
236	Parks AL, McWhirter RM, Evason K, Kelley RK. Cases of spontaneous tumor regression in hepatobiliary cancers: implications for immunotherapy? J Gastrointest Cancer. 2015;46:161–165.
237	Kim SB, Kang W, Shin SH, Lee HS, Lee SH, Choi GH, et al. Spontaneous neoplastic remission of hepatocellular carcinoma. Korean J Gastroenterol. 2015 May;65(5):312–5.
238	幸田昌樹, 杉浦 愛, 森 弘樹. 経過中に一時自然退縮を認めた肝細胞癌の1例. 浜松医療センター学術誌 2015; 9: 130-133
239	桑野哲史, 宮ケ原典, 多喜研太郎, 他. 自然退縮が疑われた肝腫瘤の1例. 診断と治療 2015; 103: 697-701
240	Matsuoka S, Tamura A, Moriyama M, Fujikawa H, Mimatsu K, Oida T, Sugitani M. Pathological evidence of the cause of spontaneous regression in a case of resected hepatocellular carcinoma. Intern Med. 2015;54:25–30.
241	Okano A, Ohana M. Spontaneous regression of hepatocellular carcinoma: its imaging course leading to complete disappearance. Case Rep Oncol 2015; 8: 94―100
242	菅本祐司, 太田拓実, 木村正幸, 他. 肥満患者の減量中に自然退縮した肝細胞癌の1例. 千葉医学 2015; 91: 59-63
243	Takeda Y, Wakui N, Asai Y, Dan N, Yamauchi Y, Ueki N, et al. Spontaneous complete necrosis of hepatocellular carcinoma: a case report and review of the literature. Oncol Lett. 2015 Apr;9(4):1520–6.
244	Tazawa, H., Fukuda, S., Fujisaki, S., Sakimoto, H., Eto, T., Moriya, T., … Nishida, T. (2015). Suggesting of spontaneous complete necrosis of hepatocellular carcinoma: Report of a case. Kanzo, 56(12), 645–654.
245	Verla-Tebit E, Rahma OE. Regression of hepatocellular carcinoma after treatment of hepatitis C: a case report. J Gastrointest Oncol. 2015 Jun;6(3):E52-4.
246	Wang Z, Ke ZF, Lu XF, Luo CJ, Liu YD, Lin ZW, Wang LT. The clue of a possible etiology about spontaneous regression of hepatocellular carcinoma: a perspective on pathology. Onco Targets Ther 2015; 8: 395-400
247	Yang SZ, Zhang W, Yuan WS, Dong JH. Recurrence of Hepatocellular Carcinoma With Epithelial-Mesenchymal Transition After Spontaneous Regression: A Case Report. Medicine (Baltimore) 2015;94:e1062.
248	Yoo YJ, Kim JH. [Spontaneous Complete Remission of Hepatocellular Carcinoma]. Korean J Gastroenterol. 2015 Dec;66(6):359-62. Korean. doi: 10.4166/kjg.2015.66.6.359. PMID: 27175457.
249	Gunasekaran SS, Emmadi R, Landers LA, Gaba RC. Regression of Hepatocellular Carcinoma Lung Metastases after Guyabano Fruit Extract Consumption. J Diet Suppl. 2016;13(3):237-44. doi: 10.3109/19390211.2015.1008613. Epub 2015 Feb 9. PMID: 25664807.
250	Heron V, Fortinsky KJ, Spiegle G, Hilzenrat N, Szilagyi A. Resected hepatocellular carcinoma in a patient with Crohn’s disease on azathioprine. Case Rep Gastroenterol 2016;10:50-6.
251	Jianxin C, Qingxia X, Junhui W, Qinhong Z. A Case of Recurrent Hepatocellular Carcinoma Acquiring Complete Remission of Target Lesion With Treatment With Traditional Chinese Medicine. Integr Cancer Ther. 2016 Epub ahead of print.
252	Kumar A, Le DT. Hepatocellular Carcinoma Regression After Cessation of Immunosuppressive Therapy. J Clin Oncol. 2016;34:e90–e92.
253	Kumar A, Le DT. Hepatocellular Carcinoma Regression After Cessation of Immunosuppressive Therapy. J Clin Oncol. 2016;34:e90–e92.
254	Luo, S. C., Wu, C. C., Jheng, S. B., & Huang, Z. Y. (2016). Spontaneous remission of hepatocellular carcinoma without any treatment. Journal of Cancer Research and Practice, 3(4), 128-131.
255	Mahmood, Shafaq & Gul, Aleesha & Saif, Tayyaba. (2016). Regression of hepatocellular carcinoma after treatment with Sofosbuvir - A case report. JPMA. The Journal of the Pakistan Medical Association. 66. 1507-1509.
256	Ooka, Y., Chiba, T., Inoue, M., Wakamatsu, T., Saito, T., Sekimoto, T., … Yokosuka, O. (2016). A case of hepatocellular carcinoma with spontaneous regression of a tumor thrombus invading the main portal trunk. Kanzo, 57(4), 178–185.
257	Pectasides E, Miksad R, Pyatibrat S, Srivastava A, Bullock A. Spontaneous regression of hepatocellular carcinoma with multiple lung metastases: A case report and review of the literature. Dig Dis Sci 2016;61:2749-54.
258	Sawatsubashi, Y., Abe, Y., Nishihara, K., Takesue, S., Nakashima, Y., & Nakano, T. (2016). A Spontaneous Complete Regression of Hepatocellular Carcinoma. The Japanese Journal of Gastroenterological Surgery, 49(6), 488–495.
259	杉浦 玄.高齢女性に発症し自然退縮した肝細胞が んの 1 例.内科 2016;117:329―331
260	Alam MA, Das D. Spontaneous Regression of Hepatocellular Carcinoma-a Case Report. J Gastrointest Cancer. 2017;48:194–197.
261	Iwatani, Y., Kuroda, D., Abe, T., Kohama, T., Urade, T., Murata, K., … Oka, S. (2017). A case of complete spontaneous necrosis with residual intrahepatic metastasis of hepatocellular carcinoma. Kanzo, 58(3), 170–175.
262	Murata, R., Kamiyama, T., Kanno, H., Yokoo, H., Orimo, T., Wakayama, K., … Taketomi, A. (2017). Spontaneous Complete Regression of a Hepatocellular Carcinoma with Hepatic Vein Tumor Thrombosis. The Japanese Journal of Gastroenterological Surgery, 50(7), 535–543.
263	Noij DP, van Der Linden PW.Spontaneous regression of hepatocellular carcinoma in a Caucasian male patient: A case report and review of the literature. Mol Clin Oncol 2017;6:225-8.
264	大山 潤，山下 航，川田秀一，他.肝細胞癌の自 然退縮と考えられた 2 例.臨床放射線 2017;62: 469―473
265	大山 潤，山下 航，川田秀一，他.肝細胞癌の自 然退縮と考えられた 2 例.臨床放射線 2017;62: 469―474
266	Sakamaki A, Kamimura K, Abe S, et al. Spontaneous regression of hepatocellular carcinoma: A mini-review. World J Gastroenterol 2017; 23 (21): 3797― 3804
267	Sano I, Kuwatani M, Sugiura R, Kato S, Kawakubo K, Ueno T, Nakanishi Y, Mitsuhashi T, Hirata H, Haba S, Hirano S, Sakamoto N. Hepatobiliary and Pancreatic: A rare case of a well-differentiated neuroendocrine tumor in the bile duct with spontaneous regression diagnosed by EUS-FNA.
268	山口晃典，藪崎哲史，加藤扶美，他.肝細胞癌の自 然退縮と考えられた 4 症例.臨床放射線 2017;62: 781―789
269	山口晃典，藪崎哲史，加藤扶美，他.肝細胞癌の自 然退縮と考えられた 4 症例.臨床放射線 2017;62: 781―789
270	山口晃典，藪崎哲史，加藤扶美，他.肝細胞癌の自 然退縮と考えられた 4 症例.臨床放射線 2017;62: 781―789
271	山口晃典，藪崎哲史，加藤扶美，他.肝細胞癌の自 然退縮と考えられた 4 症例.臨床放射線 2017;62: 781―789
272	El-Badrawy A, Abd El-Wahab R, Emarah Z, Eisa N. Rapid Spontaneous Regression of Diffuse Large B-Cell Lymphoma after Fine Needle Aspiration Cytology: A Case Report. Clin Oncol. 2018; 3: 1455.
273	Goto Y, Uchino Y, Sasaki S, et al. Complete spontaneous necrosis of hepatocellular carcinoma accompanied by portal vein tumor thrombosis: A case report. Int J Surg Case Rep 2018; 44: 220―225
274	Koya Y, Suzuki T, Tai M, Ichii O, Matsuhashi N, Ejiri Y, et al. Spontaneous regression of hepatocellular carcinoma with portal vein tumor thrombus. Case Rep Gastroenterol 2018;12:411-9.
275	Lee J, Lee N, Yoon K, et al. Cystic degeneration of hepatocellular carcinoma mimicking mucinous cystic neoplasm. Korean J Gastroenterol 2019; 73: 303―307
276	Taniai T, Shirai Y, Shiba H, Sakamoto T, Furukawa K, Yanaga K. Spontaneous pathological complete regression of hepatocellular carcinoma. Case Rep Gastroenterol 2018;12:653-9.
277	Taniguchi, M., Sakuma, Y., Koizumi, M., Sasanuma, H., Lefor, A. K., & Sata, N. (2018). Spontaneous Complete Necrosis of Hepatocellular Carcinoma—A Case Report—. Nihon Rinsho Geka Gakkai Zasshi (Journal of Japan Surgical Association), 79(9), 1922–1927.
278	Alhatem, A., Badeti, S., Liu, C., Liu, D., & Cai, D. (2019). Spontaneous Regression of Hepatic Diffuse Large B-cell Lymphoma in HIV and HCV Positive Patient: A Novel Case Study. Int J Cancer Clin Res, 6, 127.
279	M.B.Y. Chohan, N. Taylor, C. Coffin, K.W. Burak, O.F. Bathe, Spontaneous regression of hepatocellular carcinoma and review of reports in the published English literature, Case Rep. Med. 2019 (2019), 9756758.
280	Koichi Fujikawa, Ken Fujishiro, Harufumi Makino, et al. Spontaneous regression of multiple hepatocellular carcinoma and peritoneal disseminations. Rinsho Geka 2019; 74: 763-767
281	Masashi Hirota, Hiroki Murakami, Koichi Omoto, et al. A case of hepatocellular carcinoma with spontaneous regression caused by temperance and non-smoking. Surgery 2019; 81: 284-287
282	Kim, J.O.; Kim, C.A. Abscopal Resolution of a Hepatic Metastasis in a Patient with Metastatic Cholangiocarcinoma Following Radical Stereotactic Body Radiotherapy to a Synchronous Early Stage Non-small Cell Lung Cancer. Cureus 2019, 11, e4082.
283	Kawaguchi T, Nakano D, Okamura S, et al. Spontaneous regression of hepatocellular carcinoma with reduction in angiogenesis-related cytokines after treatment with sodium-glucose cotransporter 2 inhibitor in a cirrhotic patient with diabetes mellitus. Hepatology Research 2019; 49: 479―486
284	Lee J, Lee N, Yoon K, et al. Cystic degeneration of hepatocellular carcinoma mimicking mucinous cystic neoplasm. Korean J Gastroenterol 2019; 73: 303―307
285	Yoshida, T., Yoshida, S., Yamagishi, T., Yamane, H., Matsumoto, T., Kobayashi, A., … Tominaga, M. (2019). Clear Cell Type of Hepatocellular Carcinoma with a Slow Progression Accompanied by Natural Necrosis. The Japanese Journal of Gastroenterological Surgery, 52(9), 513–520.
286	Arjunan, V., Hansen, A., Deutzmann, A., Sze, D. Y., & Dhanasekaran, R. (2021). Spontaneous Regression of Hepatocellular Carcinoma: When the Immune System Stands Up to Cancer. Hepatology (Baltimore, Md.), 73(4), 1611-1614.
287	Arjunan, V., Hansen, A., Deutzmann, A., Sze, D. Y., & Dhanasekaran, R. (2021). Spontaneous Regression of Hepatocellular Carcinoma: When the Immune System Stands Up to Cancer. Hepatology (Baltimore, Md.), 73(4), 1611-1614.
288	Arjunan, V., Hansen, A., Deutzmann, A., Sze, D. Y., & Dhanasekaran, R. (2021). Spontaneous Regression of Hepatocellular Carcinoma: When the Immune System Stands Up to Cancer. Hepatology (Baltimore, Md.), 73(4), 1611-1614.
289	Arjunan, V., Hansen, A., Deutzmann, A., Sze, D. Y., & Dhanasekaran, R. (2021). Spontaneous Regression of Hepatocellular Carcinoma: When the Immune System Stands Up to Cancer. Hepatology (Baltimore, Md.), 73(4), 1611-1614.
290	Costa-Santos, M. P., Gonçalves, A., Ferreira, A. O., & Nunes, J. (2020). Spontaneous regression of hepatocellular carcinoma: myth or reality? BMJ Case Reports CP, 13(2), e233509.
291	Hokkoku D, Morimoto O, Takiuchi D, Wada R, Ikeshima R, Wada N, Munakata K, Akamaru Y, Ota H, Shibata K, Ohashi H. [A Case Report of Hepatocellular Carcinoma with Complete Spontaneous Regression]. Gan To Kagaku Ryoho. 2020 Dec;47(13):2385-2387. Japanese.
292	Muroya, D., Sato, T., Sakai, H., Hisaka, T., Akagi, Y., & Okuda, K. (2021). Spontaneous regression of lung metastases in hepatocellular carcinoma: A case report. International Journal of Surgery Case Reports, 78, 378-381.
293	Nakamoto R, Okuyama C, Oka S. Complete Spontaneous Regression of Hepatosplenic T-Cell Lymphoma After Surgical Biopsy. Clin Nucl Med. 2020 Feb;45(2):e88-e91.
294	Ohmatsu, K.; Hashimoto, Y.; Kawanishi, M.; Ishii, Y.; Kono, S.; Kuribayashi, S.; Ariizumi, S.; Karasawa, K. Abscopal complete regression of hepatocellular carcinoma with multiple pleural metastases. Int. Cancer Conf. J. 2021, 10, 54–58.
295	Onishi, Y., Kusumoto, M., Motoi, N., Hiraoka, N., Sugawara, S., Itou, C., & Sone, M. (2021). Natural history of epithelioid hemangioendothelioma of the liver: CT findings of 15 cases. Academic Radiology, 28(6), 778-782.
296	Onishi, Y., Kusumoto, M., Motoi, N., Hiraoka, N., Sugawara, S., Itou, C., & Sone, M. (2021). Natural history of epithelioid hemangioendothelioma of the liver: CT findings of 15 cases. Academic Radiology, 28(6), 778-782.
297	Onishi, Y., Kusumoto, M., Motoi, N., Hiraoka, N., Sugawara, S., Itou, C., & Sone, M. (2021). Natural history of epithelioid hemangioendothelioma of the liver: CT findings of 15 cases. Academic Radiology, 28(6), 778-782.
298	Onishi, Y., Kusumoto, M., Motoi, N., Hiraoka, N., Sugawara, S., Itou, C., & Sone, M. (2021). Natural history of epithelioid hemangioendothelioma of the liver: CT findings of 15 cases. Academic Radiology, 28(6), 778-782.
299	Onishi, Y., Kusumoto, M., Motoi, N., Hiraoka, N., Sugawara, S., Itou, C., & Sone, M. (2021). Natural history of epithelioid hemangioendothelioma of the liver: CT findings of 15 cases. Academic Radiology, 28(6), 778-782.
300	Onishi, Y., Kusumoto, M., Motoi, N., Hiraoka, N., Sugawara, S., Itou, C., & Sone, M. (2021). Natural history of epithelioid hemangioendothelioma of the liver: CT findings of 15 cases. Academic Radiology, 28(6), 778-782.
301	Raufi, A. G., May, M., Greendyk, R. A., Iuga, A., Ahmed, F., Mansukhani, M., & Manji, G. A. (2020). Spontaneous Regression and Complete Response to Immune Checkpoint Blockade in a Case of High-Grade Neuroendocrine Carcinoma. JCO Precision Oncology, (4), 1006–1011.
302	坂本愛子，高木慎太郎，福原崇之，他.自然壊死肝 細胞癌の 2 例.広島医学 2020;73:573―576
303	坂本愛子，高木慎太郎，福原崇之，他.自然壊死肝 細胞癌の 2 例.広島医学 2020;73:573―577
304	Sonbare D, J, Bandi R, Sharma V, Cacciarelli T, Shaikh O, S: Spontaneous Regression of Advanced Hepatocellular Carcinoma. Case Rep Gastroenterol 2020;14:491-496.
305	Joseph W. Franses, Irun Bhan, Amaya Pankaj, David T. Ting, Vikram Deshpande, and Kenneth Tanabe: Spontaneous Immune-Mediated Regression of Hepatocellular Carcinoma With High Tumor Mutational Burden. JCO Precision Oncology 2021:5, 1040-1043
306	Kakuta, N., Yasumoto, T., Yoshida, Y., Tsuda, M., Miyazaki, A., Miyamoto, S., ... & Naito, M. (2021). Spontaneous regression of lung metastases after transarterial chemoembolization for hepatocellular carcinoma. Radiology Case Reports, 16(6), 1530-1534.
307	Kimura T, Goi T, Yokoi S, Ohnishi K, Togawa T, Iida A, Ishida M, Sato Y. Possible spontaneous regression of hepatocellular carcinoma with unique histopathological features confirmed by surgical resection: a case report. Surg Case Rep. 2021 Jul 13;7(1):162. doi: 10.1186/s40792-021-01246-z. PMID: 34255193; PMCID: PMC8276907.
308	Liu Z, Zou JW, Wang WL, Li Z. Spontaneous regression of recurrent hepatocellular carcinoma with multiple lung metastases. J Cancer Res Ther. 2020;16(7):1710-1713.
309	Obu, M., Ishii, K., Oki, Y., Fujiya, M., Yonemoto, T., Takahashi, T., … Azemoto, R. (2022). A case of spontaneous regression of a rapidly-grown hepatocellular carcinoma. Kanzo, 63(2), 51–61.
310	Tanaka, Y., Asai, S., Hashimoto, A., & Shinzato, I. (2020). Hepatic Hodgkin Lymphoma Presenting as Solitary Hepatic Mass Following Other Iatrogenic Immunodeficiency-Associated Lymphoproliferative Disorder in a Patient With Rheumatoid Arthritis. Journal Of Medical Cases, 12(2), 79-83.
311	Shapiro SL. Spontaneous regression of cancer. Eye Ear Nose Throat Mon. 1967 Oct;46(10):1306-10. PMID: 6076351.
312	Lokich JJ, Brooks JR. Disappearance of disseminated pancreatic carcinoma with combined chemotherapy. Ann Surg. 1973;177:13–14.
313	Eidemiller LR, Fletcher WS, Dennis DL, et al. . Spontaneous remission of proven cancer. Northwest Med 1971;70:539–43.
314	Tchertkoff V, Hauser AD. Carcinoma of head of pancreas with spontaneous regression. N Y State J Med 1974;74:1814–7.
315	Gunn HD, Cann SAH, Gunn HD, et al. . Spontaneous regression of pancreatic cancer. 2016.
316	Chin KM, Chan CY, Lee SY. Spontaneous regression of pancreatic cancer: a case report and literature review. Int J Surg Case Rep. 2018;42: 55–59.
317	Sreevathsa MR, Koirala N (2018) Three decades of survival in Pancreatic Neuroendocrine Tumor with Unresectable Liver Metastases. Ann Pancreat Disord Treatm 2(1): 002-006.
318	Saade Lemus P, Anderson K, Smith M, Bullock A. Spontaneous regression of pancreatic cancer with liver metastases. BMJ Case Rep. 2019 May 31;12(5):e229619.
319	Ibrahimi S, Mukherjee S, Alhyari L, Rubin E, Aljumaily R. Spontaneous Regression of Metastatic Pancreatic Cancer: A Role for Recurrent Inflammation. Pancreas. 2019 Jan;48(1):e4-e6.
320	Kawaguchi S, Ohtsu T, Enokida K, Terada S, Endo S, Shirane N, Kanemoto H, Taku K, Muramatsu A. [A case of poorly differentiated pancreatic adenocarcinoma with spontaneous regression wherein retrospective rim enhancement findings were important for diagnosis]. Nihon Shokakibyo Gakkai Zasshi. 2021;118(4):358-365. Japanese. doi: 10.11405/nisshoshi.118.358. PMID: 33840717.
321	Sroujieh AS. Spontaneous regression of intestinal malignant melanoma from an occult primary site. Cancer. 1988 Sep 15;62(6):1247-50.
322	Nagashima R, Takeda H, Maeda K, et al. Regression of duodenal mucosa-associated lymphoid tissue lymphoma after eradication of Helicobacter pylori. Gastroenterology 111: 1674-1678, 1996.
323	Rayson D, Pitot HC, Kvols LK. Regression of metastatic carcinoid tumor after valvular surgery for carcinoid heart disease. Cancer. 1997 Feb 1;79(3):605-11. PMID: 9028374.
324	Horiuchi, T., Nomura, J., Okuda, M., & Ichinohasama, R. (2003). Abscopal effect of small intestinal NK/T-cell lymphoma. Rinsho Ketsueki, 44(9), 940–945.
325	Makino Y, Suzuki H, Nishizawa T, Kameyama K, Hisamatsu T, Imaeda H, Mukai M, Hibi T. Ileal Mucosa-Associated Lymphoid Tissue (MALT) Lymphoma with a Large-Cell Component That Regressed Spontaneously. Gut Liver. 2010 Mar;4(1):117-21.
326	Hayashi, H., Onishi, Y., Mitsuoka, H., Ogura, T., Maeda, M., Nishigami, T., & Harada, M. (2013). Regression of Follicular Lymphoma of the Duodenum Following Eradication of H. pylori Infection. Internal Medicine, 52(23), 2611–2614.
327	Tanaka, K., Ohtsuka, M., Shimizu, H., Yoshidome, H., Kato, A., Furukawa, K., Yoshitomi, H., Kishimoto, T., Nakatani, Y., & Miyazaki, M. (2014). Spontaneous Regression of Duodenal Cancer with a Metastatic Liver Cancer. The Japanese Journal of Gastroenterological Surgery, 47, 11-17.
328	Sasaki J, Kurihara H, Nakano Y, Kotani K, Tame E, Sasaki A. Apparent spontaneous regression of malignant neoplasms after radiography: Report of four cases. Int J Surg Case Rep. 2016;25:40-3. doi: 10.1016/j.ijscr.2016.05.049. Epub 2016 May 31.
329	Hori S, Tachihara M, Tamura D, Kobayashi K, Nakata K, Kamiryo H, Sakai Y, Itoh T, Hirose T, Nishimura Y. Spontaneous Regression of Epithelioid Angiosarcoma in a Young Woman. Intern Med. 2017 Dec 15;56(24):3333-3339. doi: 10.2169/internalmedicine.6754-15. Epub 2017 Oct 11.
330	Tanaka Y, Ishihara M, Miyoshi H, Hashimoto A, Shinzato I, Ohshima K. Spontaneous regression of diffuse large B-cell lymphoma in the small intestine with multiple lymphadenopathy. J Clin Exp Hematol. 2019;59(1):17-21.
331	Everson TC, Cole WH (1966) Spontaneous regression of cancer. WB Saunders, Philadelphia, PA
332	Boyd W (1966) The spontaneous regression of cancer. Charles C. Thomas, Springfield, IL
333	Fergeson JO, Black BM (1954) Disappearance, probably spontaneous of locally inoperable carcinoma of the descending colon: report of case. Mayo Clin Proc 29:407–410
334	Everson TC, Cole WH (1966) Spontaneous regression of cancer. WB Saunders, Philadelphia, PA
335	Everson TC, Cole WH (1966) Spontaneous regression of cancer. WB Saunders, Philadelphia, PA
336	Jerry LM, Challis EB. Rakel RE. Oncology, Textbook of Family Practice, 19843rd edn (pg. 1061-81)
337	Brown CH. Regression of metastatic lesions: report of two cases. Am Pract (Clin Pediatr) 1961;12:655–6.
338	Brunschwig A (1963) Spontaneous regression of cancer. Surgery 53:423–431
339	Mayo CW (1963) Tumor clinic conference. Cancer Bull 15:78–79
340	Fullerton JM, Hill RD. Spontaneous regression of cancer. Br Med J 1963;21:1589–90.
341	Rankin GB, Brown CH, Crile G Jr (1965) Spontaneous regression of hepatic metastases from a carcinoma of the colon: 10-year follow up of a patient with familial polyposis. Ann Surg 162:156–159
342	Margolis J, West D (1967) Spontaneous regression of malignant disease: report of three cases. J Am Geriatr Soc 15:251–253
343	Synder W, Clark R, Rubini J. Long-term survival of mother and son with widespread metastatic adenocarcinoma of colon. Cancer 1968;21:129–33.
344	Synder W, Clark R, Rubini J. Long-term survival of mother and son with widespread metastatic adenocarcinoma of colon. Cancer 1968;21:129–33.
345	Weinstock C. Notes on “spontaneous” regression of cancer. J Am Soc Psycosom Dent Med 1977;24:106–10.
346	Meares A. Regression of cancer of the rectum after intensive meditation. Med J Aust 1979;2:539–40.
347	Glasser M, Rosenberg MZ, Gaito R. Widespread adenocarcinoma of the colon with survival of 28 years. JAMA 1979;241:2542–3.
348	Beechey RT, Edwards BE, Kellands CH. Adenocarcinoma of the colon: an unusual case. Med J Aust 1986;144:211–3.
349	Tominaga, K., Arai, J., Imamura, Y., Ohta, A., Sato, K., Tada, T., … Nagao, J. (1999). A case of colonic early carcinoma suspected autoamputation. Progress of Digestive Endoscopy(1972), 55(2), 96–97.
350	Wadsworth SJ, Davies CW, Gray W, Gleeson FV. Spontaneous regression of pulmonary metastases demonstrated by CT. Br J Radiol 1999 Mar;72(855):304e7.
351	Okamura S, Katsuhiko H, Satoh T. Regression of rectal mucosa-associated lymphoid tissue lymphoma unrelated to Helicobacter pylori. Ann Intern Med. 2000 Feb 1;132(3):247.
352	Kamesui T, Munemoto Y, Fujisawa K, Kasahara Y, Mitsui T, Asada Y, Iida Y, Miura S, Fujisawa M, Tsukioka N. A case of spontaneous dislodging of early colon cancer. Gastroenterol Endosc. 2000;42:1218–22.
353	Takenaka R, Tomoda J, Sakata T, et al. Mucosa-associated lymphoid tissue lymphoma of the rectum that regressed spontaneously. J Gastroenterol Hepatol 2000;15:331-335.
354	Ikuta, S. I., Miki, C., Ookura, E., Tonouchi, H., & Kusunoki, M. (2002). Spontaneous regression of a metastatic liver tumor: report of a case. Surgery today, 32(9), 844-848.
355	Abdelrazeq AS, Lund JN, Leveson SH (2005) Spontaneous regression of peritoneal carcinomatosis from a rectal cancer. Eur J Gastroenterol Hepatol 17:1421–1423
356	Satoshi Itano, Norihiko Terada, Sadayuki Horiki et al .: A case of anorectal malignant melanoma falling off spontaneously and surviving over two years. Surgical treatment 2006; 94: 967-969
357	Tomiki Y, Gonda H, Seki E, et al : Spontaneous decapitation of a small colorectal cancer : follow-up of the spontaneous course. Endoscopy 2007 ; 39 : 290-291
358	Kochi, M., Yamake, H., Kaiga, T., Ookubo, R., Fujii, M., & Takayama, T. (2008). Spontaneous regression of advanced colon cancer—case report—. Nihon Rinsho Geka Gakkai Zasshi (Journal of Japan Surgical Association), 69(7), 1717–1720.
359	Bir, A. S., Fora, A. A., Levea, C., & Fakih, M. G. (2009). Spontaneous regression of colorectal cancer metastatic to retroperitoneal lymph nodes. Anticancer research, 29(2), 465-468.
360	Sakamoto S, Fu K, Kobayashi O, Matsuyama S, Miyazaki A, Ogura K, et al. Spontaneous complete regression of a rectal cancer. Endoscopy. 2009;41: 910–2.
361	Shimizu H, Kochi M, Kaiga T, Mihara Y, Fujii M, Takayama T. A case of spontaneous regression of advanced colon cancer. Anticancer Res. 2010;30: 2351–4.
362	Sakuma, A., Yoshimatsu, K., Yokomizo, H., Osawa, G., Shimazaki, A., Matsumoto, A., … Ogawa, K. (2011). Radical Excision of Suspected Recurrent Sigmoid Colon Cancer after Spontaneous Regression. Nippon Daicho Komonbyo Gakkai Zasshi, 64(2), 83–87.
363	Nakashima M, Hori K, Kimura Y, Hayashi K, Yokomizo H, et al. A case of spontaneous regression of colon cancer. The journal of the Japan Surgical Association. 2012;73(6):1482–5.
364	Ann D. Flynn, MD, Jose M. Azar, MD, Michael V. Chiorean, MD, Spontaneous Regression of Colonic Lymphoma Following Infliximab and Azathioprine Withdrawal in Patients With Crohn’s Disease, Inflammatory Bowel Diseases, Volume 19, Issue 5, 1 April 2013, Pages E69–E70,
365	Ann D. Flynn, MD, Jose M. Azar, MD, Michael V. Chiorean, MD, Spontaneous Regression of Colonic Lymphoma Following Infliximab and Azathioprine Withdrawal in Patients With Crohn’s Disease, Inflammatory Bowel Diseases, Volume 19, Issue 5, 1 April 2013, Pages E69–E70,
366	Nakamura F, Sakamoto T, Nakajima T, Saito Y, Taniguchi H, Matsuda T. A case of rectal tumor in which the shape altered with regression in short period. BMC Gastroenterol. 2013;13:146.
367	Sekiguchi M, Mtsumoto N. Spontaneously disappearing colon cancer. Japan Gastroenterological Endoscopy Society. 2013;25:84–93.
368	Kihara K, Fujita S, Ohshiro T, Yamamoto S, et al. Spontaneous regression of colon cancer. Jpn J Clin Oncol. 2015;45(1):111–4.
369	Mitchell, A., & Bendavid, Y. (2014). Medullary colon cancer presenting with total necrosis of all regional lymph node metastases: morphologic description of a presumed immune-mediated event. Diagnostic Pathology, 9(1), 1-5.
370	Sewpaul A, Bargiela D, James A, Johnson SJ, French JJ. Spontaneous Regression of a Carcinoid Tumor following Pregnancy. Case Rep Endocrinol. 2014;2014:481823.
371	Kihara, K., Fujita, S., Ohshiro, T., Yamamoto, S., & Sekine, S. (2015). Spontaneous regression of colon cancer. Japanese Journal of Clinical Oncology, 45(1), 111-114.
372	Serizawa M, Kawarabayashi N. A case of spontaneous regression of transverse colon cancer. Gastroenterol Endosc. 2015;57(7):1490–5.
373	Ito, S., Kobayashi, O., Ohta, K., Kojima, T., Hashimoto, S., Miyoshi, Y., … Watanabe, S. (2016). Spontaneous regression of ascending colon cancer : Case report. Progress of Digestive Endoscopy, 88(1), 152–153.
374	Nemésio, R. A., Martins, R., Cipriano, M. A., Tralhão, J. G., & e Sousa, F. C. (2016). Spontaneous regression of colorectal liver metastases. Archives in Cancer Research, 4(1), 0-0.
375	Chida K, Nakanishi K, Shomura H, et al. Spontaneous regression of transverse colon cancer: a case report. Surgical Case Reports. 2017;3:65.
376	Matsuki, R., Sugiyama, M., Yoshiike, S., Shibahara, J., Kogure, M., Yokoyama, M., ... & Mori, T. (2018). Spontaneous regression of colorectal liver metastasis. Clinical Journal of Gastroenterology, 11(4), 263-267.
377	Chuang, C.-H.; Hsu, J.-F.; Shen, Y.-T.; Yang, C.-J. Regression of a metastatic lung mass after receiving whole brain irradiation: Can the abscopal effect cross the blood-brain barrier? Asia Pac. J. Clin. Oncol. 2018, 14, e548–e550.
378	Yoshida K, Sawaya M, Mikami T, et al. A case of transverse colon cancer that underwent spontaneous regression after biopsy. Gastroenterol Endosc. 2018;60:2303–9.
379	Fukutomi, T., Sato, A., Chiba, H., & Itakura, Y. (2019). A Case of Spontaneous Regression of Advanced Transverse Colon Cancer. Nihon Rinsho Geka Gakkai Zasshi (Journal of Japan Surgical Association), 80(8), 1501–1507.
380	Karakuchi N, Shimomura M, Toyota K, Hinoi T, Yamamoto H, Sadamoto S, Mandai K, Egi H, Ohdan H, Takahashi T. Spontaneous regression of transverse colon cancer with high-frequency microsatellite instability: a case report and literature review. World J Surg Oncol. 2019 Jan 15;17(1):19.
381	Kawakita I, Takeda K, Tanaka Y, et al. Spontaneous regression of ascending colon cancer. Jpn J Gastroenterol Surg. 2019;52:106–11.
382	Tanaka, F., Hiraki, M., Yamada, K., Tominaga, N., Ikeda, O., Kido, S., … Kitahara, K. (2019). A Case of Spontaneous Regression of Sigmoid Colon Cancer after Local Injection for Non-lifting Sign Evaluation. Nihon Rinsho Geka Gakkai Zasshi (Journal of Japan Surgical Association), 80(2), 368–372.
383	Utsumi T, Miyamoto S, Shimizu T, et al. Spontaneous regression of mismatch repair-deficient colorectal cancers: a case series. Dig Endosc 2020. Epub 2020/05/18.
384	Utsumi T, Miyamoto S, Shimizu T, et al. Spontaneous regression of mismatch repair-deficient colorectal cancers: a case series. Dig Endosc 2020. Epub 2020/05/18.
385	Utsumi T, Miyamoto S, Shimizu T, et al. Spontaneous regression of mismatch repair-deficient colorectal cancers: a case series. Dig Endosc 2020. Epub 2020/05/18.
386	Mehawej, J., El Helou, N., Pejovic, T., & Mhawech-Fauceglia, P. (2020). Metastatic colorectal carcinoma to one ovary with vanishing primary: A case report in an 18-year-old patient. Clin Obstet Gynecol, 6, 1-3.
387	Nishiura, B., Kumamoto, K., Akamoto, S., Asano, E., Ando, Y., Suto, H., ... & Suzuki, Y. (2020). Spontaneous regression of advanced transverse colon cancer with remaining lymph node metastasis. Surgical Case Reports, 6(1), 1-6.
388	Yokota, T., Saito, Y., Takamaru, H., Sekine, S., Nakajima, T., Yamada, M., ... & Matsuda, T. (2021). Spontaneous Regression of Mismatch Repair-Deficient Colon Cancer: A Case Series. Clinical Gastroenterology and Hepatology, 19(8), 1720-1722.
389	Yokota, T., Saito, Y., Takamaru, H., Sekine, S., Nakajima, T., Yamada, M., ... & Matsuda, T. (2021). Spontaneous Regression of Mismatch Repair-Deficient Colon Cancer: A Case Series. Clinical Gastroenterology and Hepatology, 19(8), 1720-1722.
390	Yokota, T., Saito, Y., Takamaru, H., Sekine, S., Nakajima, T., Yamada, M., ... & Matsuda, T. (2021). Spontaneous Regression of Mismatch Repair-Deficient Colon Cancer: A Case Series. Clinical Gastroenterology and Hepatology, 19(8), 1720-1722.
